# Morphology, Reproduction and Diet in Australian and Papuan Death Adders (*Acanthophis*, Elapidae)

**DOI:** 10.1371/journal.pone.0094216

**Published:** 2014-04-09

**Authors:** Richard Shine, Carol L. Spencer, J. Scott Keogh

**Affiliations:** 1 School of Biological Sciences A08, University of Sydney, Sydney, NSW, Australia; 2 Museum of Vertebrate Zoology, University of California, Berkeley, California, United States of America; 3 Evolution, Ecology and Genetics, Research School of Biology, The Australian National University, Canberra, ACT, Australia; The University of Wollongong, Australia

## Abstract

Death adders (genus *Acanthophis*) differ from most other elapid snakes, and resemble many viperid snakes, in their thickset morphology and ambush foraging mode. Although these snakes are widely distributed through Australia and Papua New Guinea, their basic biology remains poorly known. We report morphological and ecological data based upon dissection of >750 museum specimens drawn from most of the range of the genus. Female death adders grow larger than conspecific males, to about the same extent in all taxa (20% in mean adult snout-vent length,  =  SVL). Most museum specimens were adult rather than juvenile animals, and adult males outnumbered females in all taxa except *A. pyrrhus*. Females have shorter tails (relative to SVL) than males, and longer narrower heads (relative to head length) in some but not all species. The southern *A. antarcticus* is wider-bodied (relative to SVL) than the other Australian species. Fecundity of these viviparous snakes was similar among taxa (mean litter sizes 8 to 14). Death adders encompass a broad range of ecological attributes, taking a wide variety of vertebrate prey, mostly lizards (55%), frogs and mammals (each 21%; based on 217 records). Dietary composition differed among species (e.g. frogs were more common in tropical than temperate-zone species), and shifted with snake body size (endotherms were taken by larger snakes) and sex (male death adders took more lizards than did females). Overall, death adders take a broader array of prey types, including active fast-moving taxa such as endotherms and large diurnal skinks, than do most other Australian elapids of similar body sizes. Ambush foraging is the key to capturing such elusive prey.

## Introduction

Information on snake ecology and behavior has accumulated substantially over recent years. Data are now available on a much broader array of traits, revealing unsuspected complexity in dimensions such as mating systems and sociality [1]–[3] and a greater phylogenetic diversity of snake taxa than was the case a few decades ago. Nonetheless, our understanding of broad patterns in snake ecology still lags behind that for many other groups, notably lizards [4], [5]. That situation reflects logistical impediments to ecological research: many snakes are rare, often inactive, and highly cryptic, rendering them difficult to study using conventional methods. The development of miniature radio-transmitters has revolutionized this field [6], [7], but telemetry is expensive and effort-intensive, and is difficult to apply to small and slender-bodied species.

The measurement and dissection of preserved snakes in museum collections offers a simple alternative method to quantify many fundamental ecological traits. Even for rare species, considerable information can be obtained on important life history attributes such as body sizes at maturity, sexual size dimorphism, reproductive output and, because snakes ingest relatively large and whole prey items, dietary composition. The use of previously-collected museum specimens also avoids ethical and conservation issues associated with collecting and killing animals. Museum-based studies thus provide a straightforward first step towards understanding the ecological characteristics of poorly known snake taxa and can provide long-term data for temporal and geographic comparison. Importantly, such work can be conducted with simple and inexpensive equipment, and thus is suitable for parts of the world where limited funding and/or scientific infrastructure preclude other technologies.

The first extensive use of museum specimens to quantify snake ecology was based on the Australian fauna, when one of us (RS) examined >22,000 preserved specimens of 103 species within the terrestrial snake fauna of that continent and islands to the north [8], [9]. Although subsequent fieldwork has documented ecological traits in additional species, and has provided great detail about the biology of some taxa, some other Australian snake taxa have been largely neglected – including the widely-distributed and phenotypically distinctive death adders (genus *Acanthophis*). An early museum-based study described morphology, reproduction and diet of *A. antarcticus* from southeastern Australia, emphasizing the evolutionary convergence between this heavy-bodied taxon and viperids [10]. Although detailed ecological studies have been conducted on tropical death adders since that time [11]–[23], the basic morphological and ecological variables that are quantifiable from museum-based studies have not been examined for any *Acanthophis* species other than *A. antarcticus* from the mesic southeastern part of Australia. The current study addresses that gap in knowledge.

## Materials and Methods

### Operational Taxonomic Units

Taxonomic confusion remains a powerful obstacle to any overview of death adder biology. The intra-generic classification of these snakes remains uncertain, with some of the currently-recognized taxa known to be composite, and additional taxa still to be described [24], [25]. A plethora of inadequate species descriptions have confused the situation [26]. In the absence of a well-resolved phylogeny and clear species definitions, we adopted a conservative approach, accepting the risk of lumping taxa that eventually will prove to be composite, rather than splitting species that may prove to be continuous. We thus recognized six operational taxonomic units (OTUs) for our purposes: two regional populations of *Acanthophis antarcticus* (from southeastern Australia i.e. Victoria, New South Wales and southern Queensland) that together form a monophyletic clade [25], two arid-zone taxa from Western Australian deserts (*A. pyrrhus* and *A. wellsi*) that also likely are each other's closest relatives [24], [25], and two poorly-defined tropical groups that are both composite. Within this latter group, *Acanthophis praelongus* from tropical Australia probably consists of at least three taxa [24], [25] (and W. Wüster and P. Doughty, pers. comm). We treated all death adders from Papua New Guinea (PNG) as a single taxon, although they include representatives of at least two lineages (the *laevis* and *rugosus* groups) that invaded PNG separately from Australia [25], because we were unable to reliably identify many specimens. Thus, our analysis was based on two OTUs from mesic southern Australia (both referable to *A. antarcticus*), two from the Australian arid zone (*A. pyrrhus* and *A. wellsi*), one from tropical Australia (*A. “praelongus”*) and one from PNG (*A. “laevis-rugosus”*).

**Figure 1:**
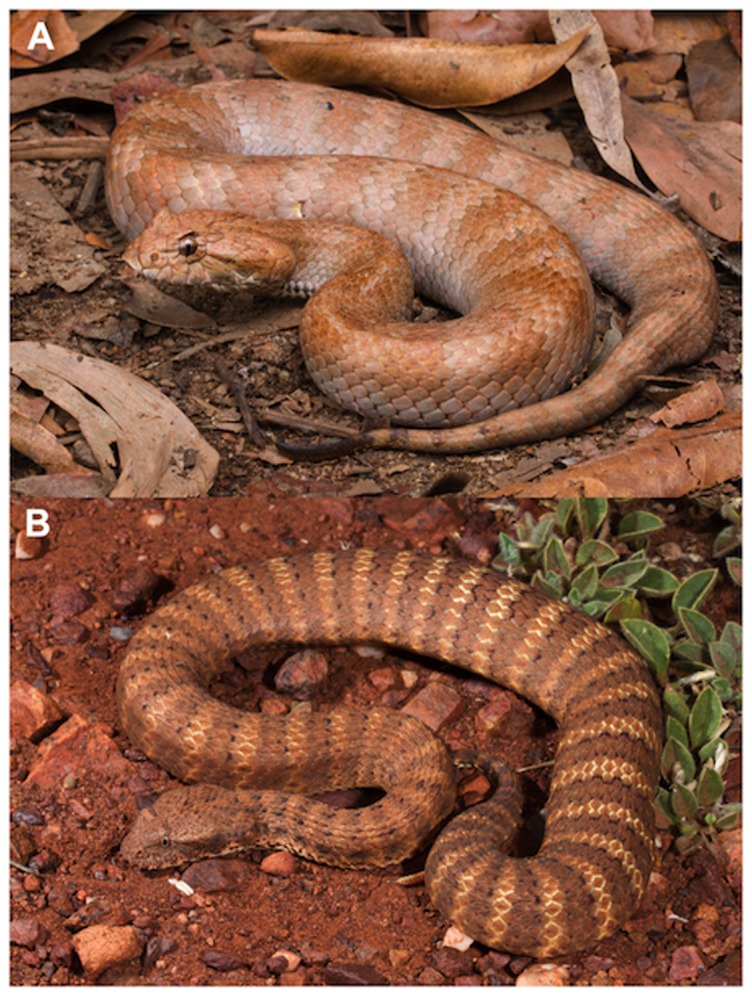
Photographs showing the diversity of body forms within *Acanthophis*. (A) tropical death adder (*Acanthophis praelongus*) from Iron Range National Park, Queensland, and (B) a desert death adder (*A. wellsi*) from Karatha, Western Australia. Photographs by Stephen Zozaya, with permission.

Although all *Acanthophis* species are heavier-bodied and larger-headed than most other elapid snakes, there is substantial variation in these morphological traits among the taxa of death adders. For example, the arid-zone species (*A. pyrrhus* and *A. wellsi*) are slender compared to the heavy-bodied southern *A. antarcticus* and the tropical *A. praelongus* (Fig. 1). Coloration also varies considerably, intra-specifically as well as inter-specifically. Like many ambush foragers that rely upon camouflage against the background, death adders show both geographic variation in dorsal color, and intra-population polymorphism in color [27], and for cases of color polymorphism in other ambush-foraging snakes see [28], [29]. In at least one species (*A. antarcticus*), an individual snake's color also varies seasonally [27].

## Methods

The three authors of this paper examined approximately 760 preserved death adders in the collections of (1) the Australian Museum (Sydney) and Queensland Museum (Brisbane), examined by RS in 1979; (2) the Queensland Museum (for specimens collected after 1979), the Western Australian Museum (Perth) and Northern Territory Museum (Darwin), examined by JSK in 1994–5; and (3) the California Academy of Science (San Francisco, CA), the B. P. Bishop Museum (Honolulu, HI) the Field Museum of Natural History (Chicago, IL), University of Texas at Arlington, the Carnegie Museum of Natural History (Pittsburgh, PA), University of Michigan Museum of Zoology (Ann Arbor, MI) and the Smithsonian (US National Museum, Washington, DC), examined by CLS between 1998–2000. We thank the museum collection managers and curators for access and for allowing us to dissect specimens, including staff of the Australian Museum (Allen Greer, Ross Sadlier), Queensland Museum (Jeanette Covacevich, Patrick Couper), Western Australian Museum (Glenn Storr, Laurie Smith), Northern Territory Museum (Paul Horner), California Academy of Science (J. Vindum, R. Drewes), Bishop Museum (A. Allison), Carnegie Museum of Natural History (S. Rogers), Field Museum of Natural History (A. Resetar), Smithsonian (R. McDiarmid, K. de Quieroz, R. Reynolds), University of Michigan Museum of Zoology (G. Schneider), and University of Texas at Arlington (J. Campbell). Table S1 lists these specimens.

The collection dates of these specimens ranged over a 100-year period, from 1896–1996. The methods used were identical, except that additional variables were recorded in later studies. The investigator first measured SVL and tail length with a ruler or cloth tape stretched along the animal's midline, and then measured the length and width of the head using calipers. Head length was taken from the tip of the nose to the quadrate-articular projection behind the lower jaw, and head width was measured at the widest part of the head. The width of the largest ventral scale at midbody was also recorded. The animal was then carefully opened along the ventral midline to reveal stomachs and hindguts (and thus, any prey items in the alimentary tract) and gonads (testes and efferent ducts in males; ovaries and oviducts in females). Males were scored as adult if the testes were turgid and/or the efferent ducts contained sperm (as indicated by opacity and distension). Females were scored as mature if they had large thickened muscular oviducts, and/or ovarian follicles >10 mm in length, following the methods of Shine (1980) [10]. If snake hindguts contained abundant insect fragments but no vertebrate remains, we tentatively identified these insect fragments as secondary items from the stomachs of ingested anurans. Invertebrates are rarely consumed as primary prey items by Australian elapid snakes, although there has been one reported exception [30]. Prey items were removed and identified using light microscopy.

Sample sizes differed among traits because some specimens could not be scored for some traits (due to damage during or after collection); also, collection data (e.g. times, specific locations) were missing for some taxa. Previously published data on southeastern Australian *A. antarcticus* from Shine's (1979) dissections (*N* = 344) [10] were included within our dataset, to maximize the power of our comparisons. Our statistical analyses were conducted with the program JMP 9.0 (SAS Institute, Cary, NC). We used contingency-table analysis to examine categorical data, logistic regression to evaluate the effects of continuous variables (e.g. body size) as well as categorical variables (e.g. sex) on categorical response variables (e.g. prey type), and ANOVA to evaluate the effects of continuous and categorical variables on continuous dependent variables (e.g. body size). Our analyses of relative body proportions used ANCOVA with the relevant body dimension as a covariate (e.g. to look at relative tail length, we used ANCOVA with tail length as the dependent variable and SVL as a covariate) but for clarity, we graph residual scores from the general linear regression between the two dimensions (e.g. from regression of tail length vs. SVL). In analyses where higher-order interaction terms were non-significant (*P*>0.05), we deleted the interaction and recalculated.

## Results

### Population Structure

**Figure 2:**
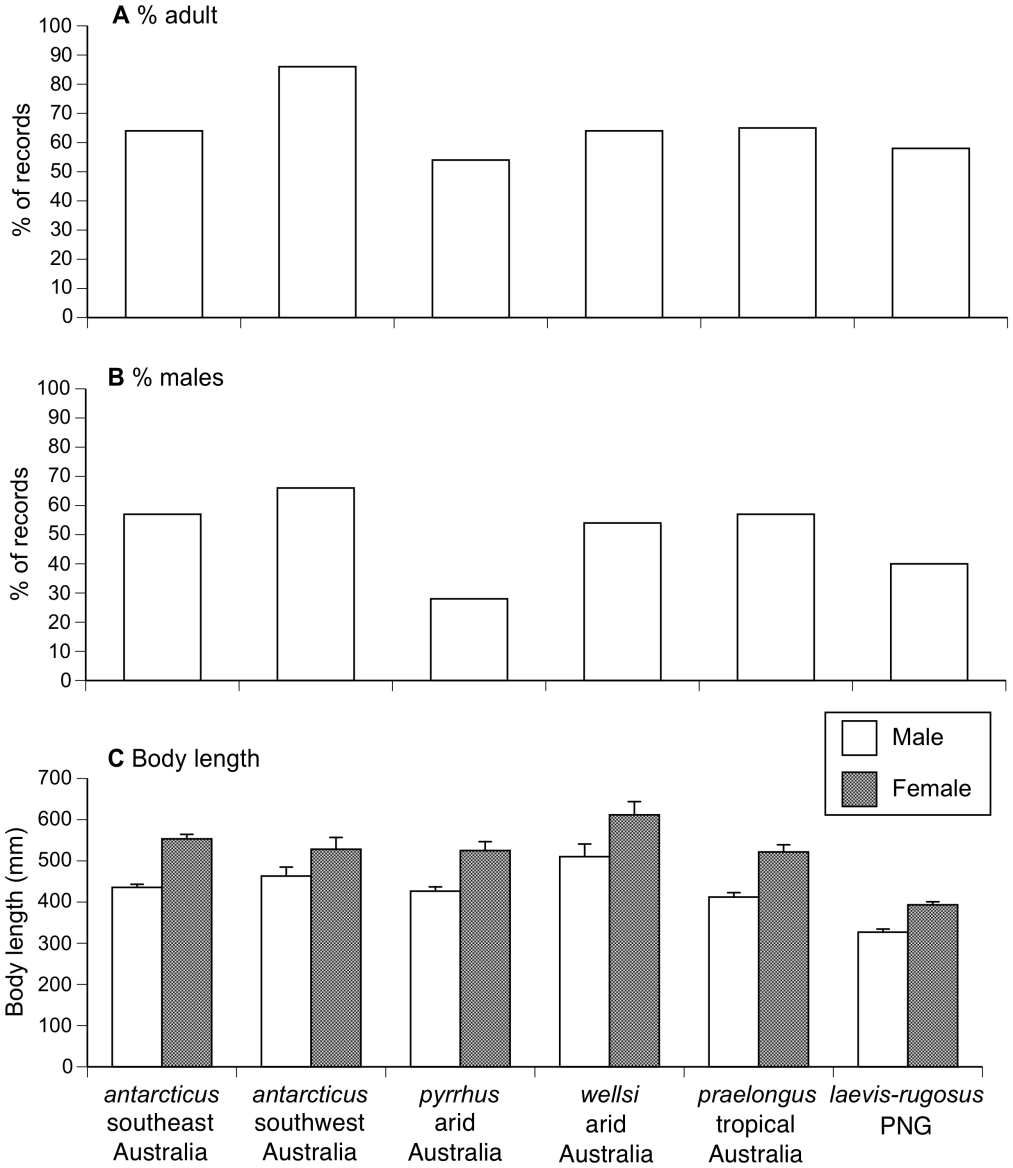
Interspecific variation in population structure and sexual size dimorphism in museum specimens of death adders (genus *Acanthophis*). The panels show (A) the proportion of juvenile animals, (B) the sex ratio of adult animals, and (C) mean adult snout-vent lengths of males and females. Graphs show mean values and associated standard errors.

The proportion of museum specimens examined that were adult rather than juvenile ranged from 54% in *A. pyrrhus* to 86% in southwestern Australian *A. antarcticus*; all other species were within the range 54–65% adults. Interspecific variation in this variable was statistically significant (χ^2^ = 13.02, df = 5, *P* = 0.023; Fig. 2A). Among adult animals, the sex ratio (% male) ranged from 28% (in *A. pyrrhus*) to 66% (southwestern Australian *A. antarcticus*: χ^2^ = 18.55, df = 5, *P* = 0.002; Fig. 2B).

### Body Sizes and Shapes

Mean adult SVLs varied among species (*F*_5,547_ = 61.22, *P*<0.0001), with females attaining average adult sizes about 20% longer than males (*F*_1,457_ = 83.18, *P*<0.0001). The degree of sexual size dimorphism did not differ significantly among species (interaction species*sex, *F*_5,457_ = 1.93, *P* = 0.09). Death adders from PNG were smaller than mainland Australian taxa, all of which had average adult SVLs around 50 cm (Fig. 2C).

**Figure 3:**
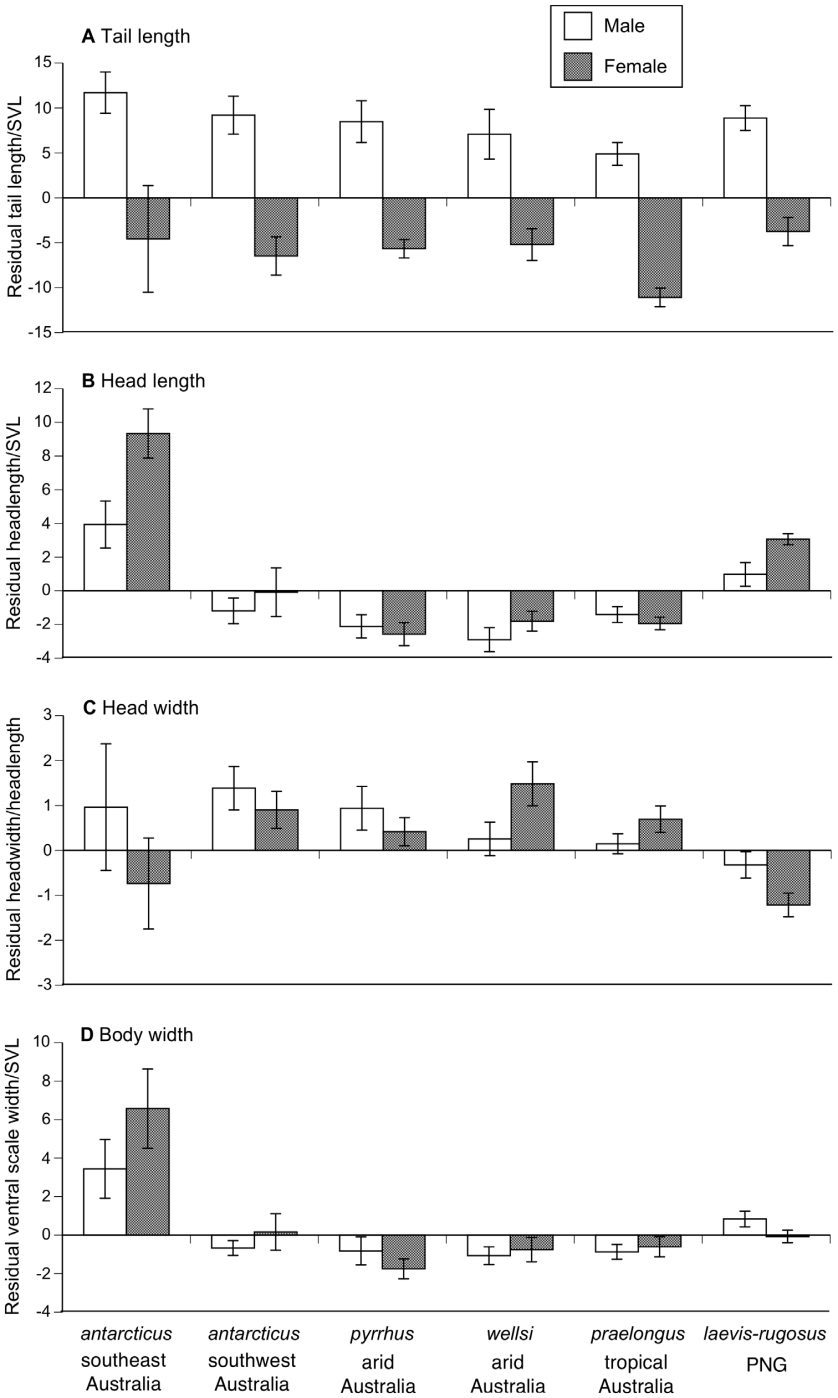
Interspecific and sexual variation in morphological traits in museum specimens of death adders (genus *Acanthophis*). The graphs show residual scores from general linear regressions to show (A) relative tail length (from the regression of tail length to snout-vent length), (B) relative head length (from the regression of head length to snout-vent length), (C) relative head width (from the regression of head width to head length), (D) relative ventral scale width (from the regression of ventral scale width to snout-vent length). Graphs show mean values and associated standard errors.

Male and female death adders differed in body proportions as well as in absolute SVL. Compared to conspecific males at the same SVL, females had shorter tails, and tail length increased more rapidly with increasing SVL in males than in females (main effect of sex, *F*_1,312_ = 4.36, *P*<0.04; interaction sex*SVL, *F*_1,312_ = 26.70, *P*<0.0001; Fig. 3A). We found no significant interspecific variation in tail length relative to SVL (main effect of species *P* = 0.14, interactions *P*>0.29).

In contrast, head length relative to SVL differed strongly among species, with relatively larger heads in *A. antarcticus* from southeastern Australia than in other taxa (Fig. 3B). Females had larger heads than conspecific males (relative to SVL) in four of the taxa we studied, but the reverse was true in *A. praelongus* and *A. pyrrhus* (Fig. 3B). Thus, statistical analysis of variation in head length showed a significant three-way interaction between species, SVL and sex (*F*_5,323_ = 8.73, *P*<0.0001).

We examined head shape by using head width as the dependent variable and head length as the covariate. Sex differences in head shape differed among taxa (interaction sex*species, *F*_5,229_ = 2.54, *P*<0.03; Fig. 3C). Females had narrower heads than males in PNG snakes, in *A. antarcticus*, and in *A. pyrrhus*. In contrast, female *A. praelongus* and *A. welllsi* had wider heads than males (Fig. 3C).

The width of the midbody ventral scales relative to SVL provides a measure of how wide-bodied a species is. Some species were wider-bodied than others, with southeastern *A. antarcticus* exceptionally wide-bodied (*F*_5,315_ = 12.14, *P*<0.0001; Fig. 3D). Overall, females tended to be wider-bodied than males at the same SVL (sex*SVL, *F*_1,303_ = 1.80, *P* = 0.024), although the reverse was true in *A. pyrrhus* and PNG snakes (Fig. 3D).

### Fecundity

**Figure 4:**
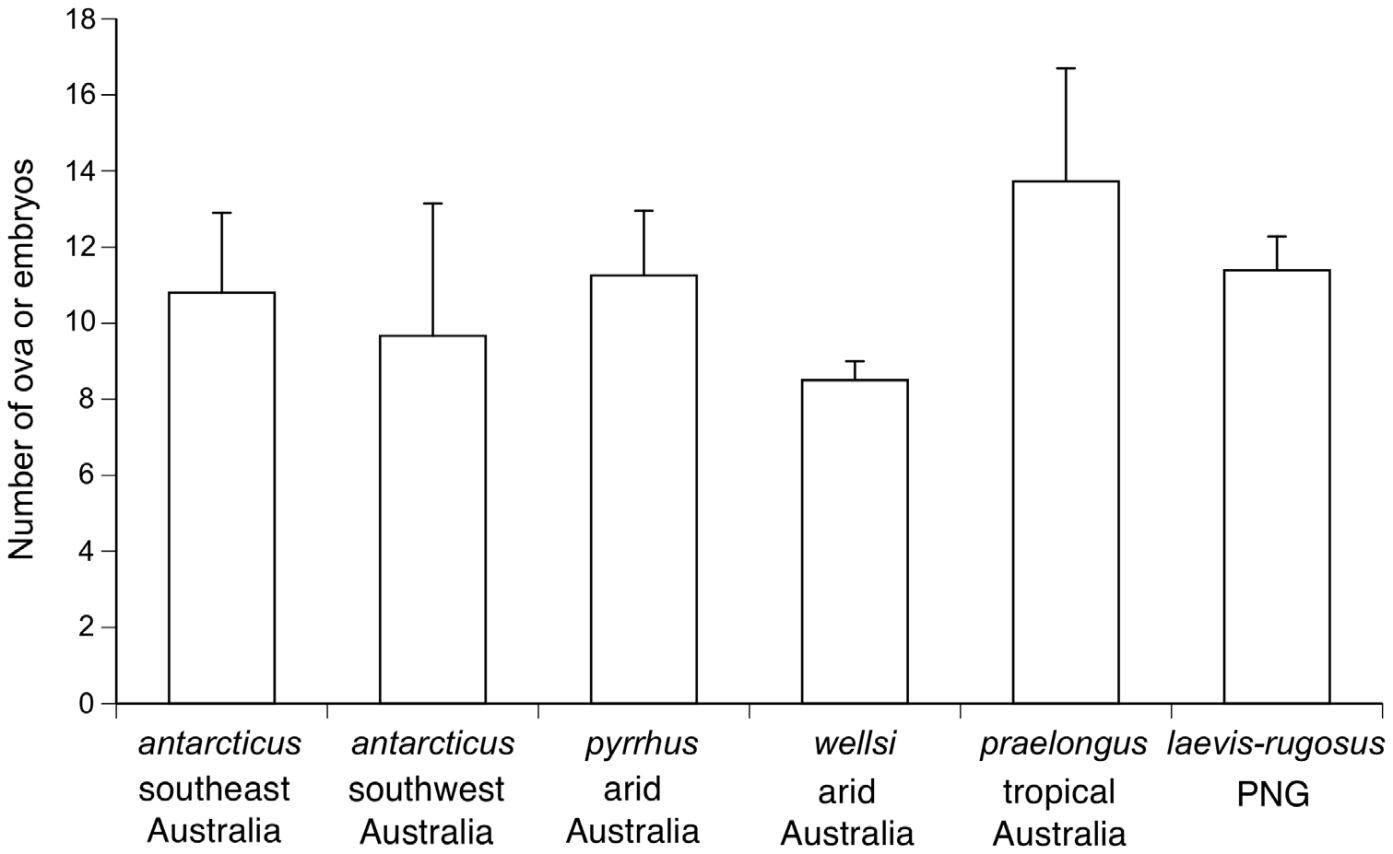
Interspecific variation in mean litter sizes of death adders (genus *Acanthophis*). Graph shows mean values and associated standard errors.

Our dissections of female death adders confirmed viviparity as the reproductive mode in all of the pregnant death adders that we examined. Litter sizes ranged from 2 to 49, with averages per species of 8.5 to 13.7 offspring (Fig. 4). Thus, the species did not differ significantly in overall mean fecundity (one-factor ANOVA, *F*_5,106_ = 0.29, *P* = 0.92).

### Dietary Composition

Death adders consumed a diverse array of vertebrates, but we found no evidence of predation on invertebrates (e.g. no cases of large intact insects in stomachs). Thus, we interpreted insect remains in hindguts as evidence of ingestion of frogs (which typically leave no identifiable tissue in the predator's hindgut). Lizards were the most common prey type (120 of 217 records), followed by frogs and mammals (*N* = 45 records each) and then birds (*N* = 7). We identified a broad range of vertebrate prey species, with high family-level diversity (5 hylid frogs, 10 agamid lizards, 2 diplodactylid lizards, 76 skinks, 2 varanids, 10 rodents, and 5 dasyurid marsupials; Table 1), with skinks by far the most important single family-level taxon. Logistic regression with prey group (frog, lizard, bird, mammal) as the dependent variable suggested that a snake's diet was affected by the predator's species (χ^2^ = 68.02, df = 15, *P* = 0.0001), its SVL (χ^2^ = 11.12, df = 3, *P* = 0.011) and its sex (χ^2^ = 11.33, df = 3, *P* = 0.01). We discuss each of these effects below.

**Table 1:** Prey items identified from dissection of museum specimens of death adders, genus *Acanthophis*.

	SE *antarcticus*	SW *antarcticus*	*pyrrhus*	*wellsi*	*praelongus*	PNG
**ANURANS**						
Unidentified spp.			1	8	9	22
**Hylidae**						
* Cyclorana* spp.					1	
* Litoria* spp.			1		2	
* Litoria latopalmata*					1	
**LIZARDS**						
Unidentified spp.			4	12	4	16
**Agamidae**						
*Amphibolurus muricatus*		1	2		1	
*Ctenophorus* spp.			2			
*Ctenophorus caudicinctus*			1			
*Diporiphora winneckei*			1			
*Intellagama lesueurii*	1					
*Physignathus gilberti*					1	
**Diplodactylidae**						
*Diplodactylus stenadactylus*			1		1	
**Scincidae**						
Unidentified spp.	3	12				3
*Carlia* spp.	1		5	2	4	
*Carlia bicarinata*						1
*Carlia foliorum*				1		
*Cryptoblepharus boutonii*						1
*Cryptoblepharus plagiocephalus*		1			2	
*Ctenotus grandis*			1			
*Ctenotus inornatus*					1	
*Ctenotus larbillardieri*		1				
*Ctenotus robustus*						
*Ctenotus taeniolatus*	1					
*Egernia* spp.		3				2
*Egernia formosa*			1			
*Egernia napoleonis*		1				
*Emoia* spp.						3
*Emoia jakati*						4
*Emoia pallidiceps*						2
*Eulamprus quoyii*	1					
*Lampropholis* spp.	2					
*Lampropholis delicata*	1					
*Lerista* spp.		1				
*Menetia greyii*	2					
*Morethia* spp.		1				
*Morethia obscura*		7				
*Sphenomorphus* spp.						3
*Sphenomorphus melanopogon*						1
**Varanidae**					2	
**BIRDS**						
Unidentified spp.	3	2		1	1	
**MAMMALS**						
Unidentified spp.	6		2	4	12	4
Unidentified rodent spp.			1			
*Mus domesticus*	4		1	2	1	
* Rattus* spp.						1
* Rattus colletti*					1	
**Dasyuridae**						
*Antechinus* spp.	1			2		
*Sminthopsis murina*					2	

#### (i) Species effect

**Figure 5:**
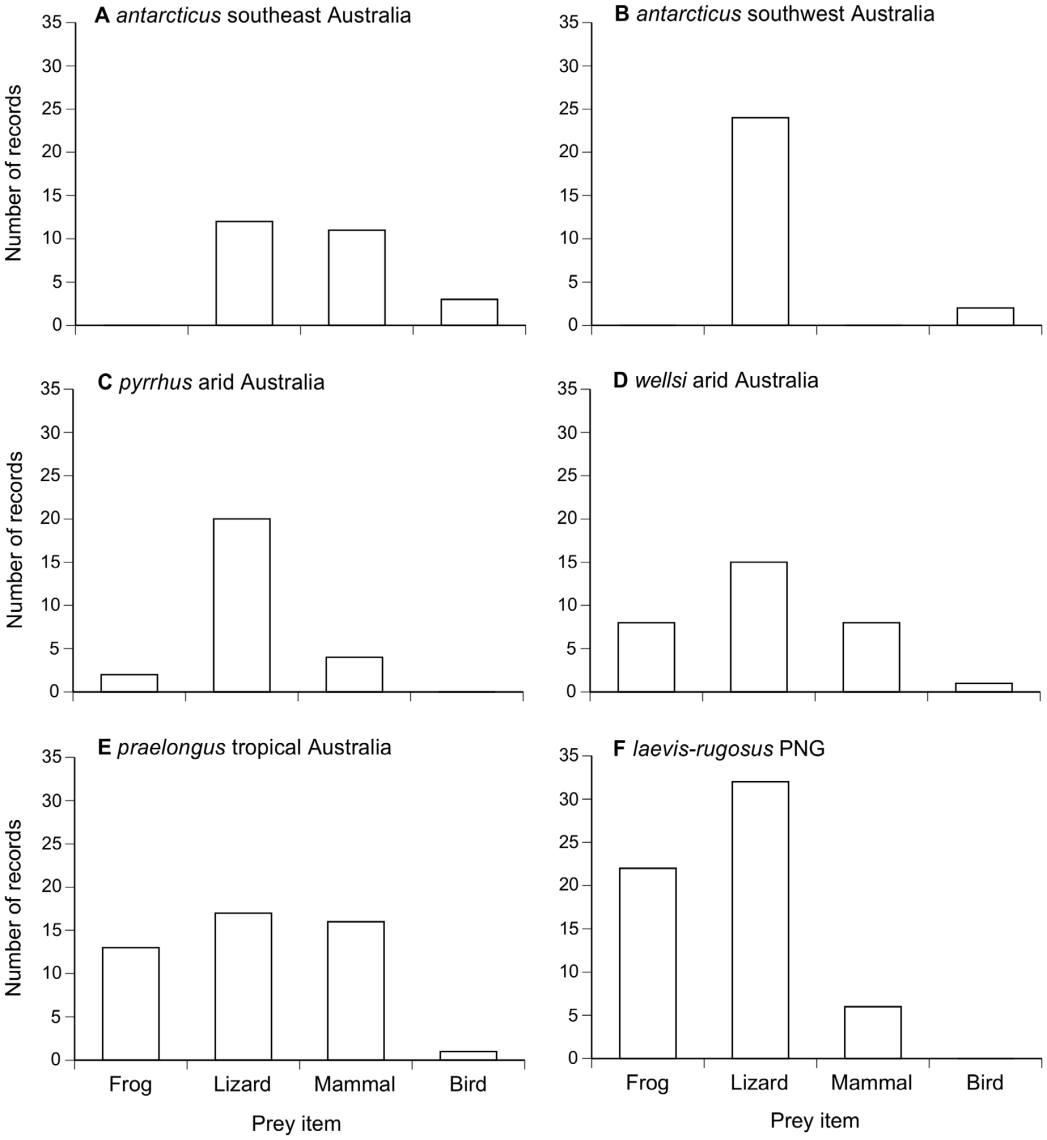
Composition of the diet in death adder species (genus *Acanthophis*). Numbers of prey are based on dissection of museum specimens.

Lizards were the most common prey type for all species, and dominated the diet of *A. pyrrhus* and southwestern *A. antarcticus*. In contrast, death adders in the tropics (PNG and northern Australia) often consumed frogs (Fig. 5). Mammals were commonly taken by *A. praelongus* and southeastern *A. antarcticus*, but not the other species. Although birds were rarely eaten, they were found in four of the six death adder taxa that we examined.

#### (ii) Body size effect

**Figure 6:**
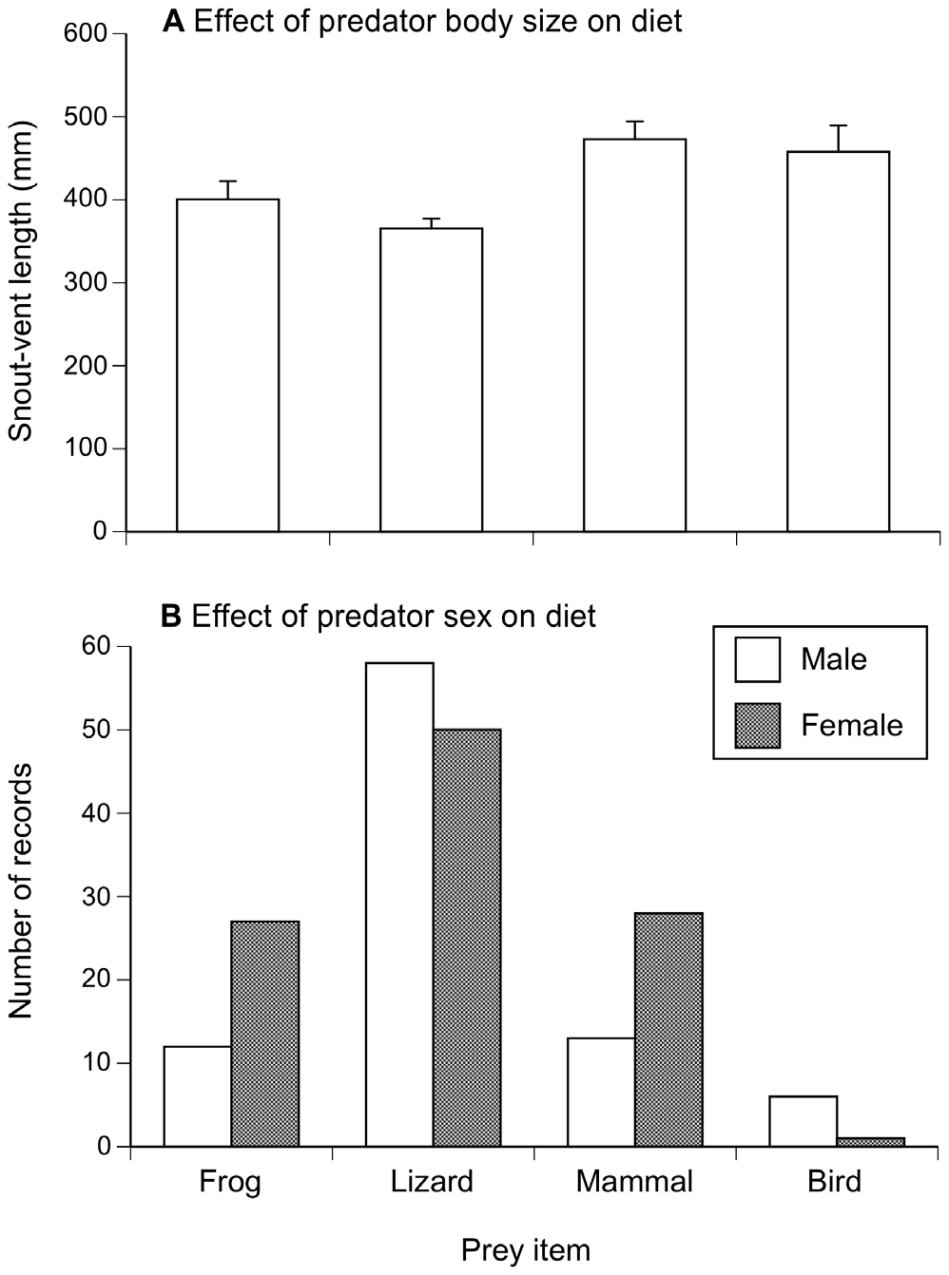
Influence of size and sex on prey consumption in *Acanthophis*. Panels show the effects of (A) body size and (B) sex on the prey types consumed by death adders. Graphs show mean values and associated standard errors.

Combining species of snakes, the average body size (SVL) of death adders that contained endothermic prey (mammals and birds) was larger than of snakes that contained ectotherms (frogs and lizards: one-factor ANOVA, *F*_3,199_ = 7.35, *P* = 0.0001; Fig. 6A).

#### (iii) Sex effect

Male death adders contained many more lizards than any other prey type, whereas females had a broader diet (Fig. 6B). The pattern was similar within species, but not statistically significant in most taxa due to low sample sizes. Among PNG adders, however, the sex divergence was significant (e.g. males consumed 6 frogs and 22 lizards, whereas females consumed 15 frogs and 9 lizards: χ^2^ = 7.43, df = 1, *P*<0.007).

Overall, we recorded identifiable prey items in 30% of male snakes, and in 33% of females. A logistic regression, with presence of prey as the dependent variable, revealed strong variation in feeding rate among species (χ^2^ = 79.59, df = 5, *P* = 0.0001), but not as a function of snake SVL (χ^2^ = 2.26, df = 1, *P* = 0.13) or sex (χ^2^ = 1.62, df = 1, *P* = 0.20). The proportion of animals containing food was lower in southeastern *A. antarcticus*, and in PNG snakes, than in the other taxa. Given that some collectors may have retained animals for longer periods in captivity prior to killing them, and that the specimens were collected over a 100-year time period, these differences are difficult to interpret.

## Discussion

Although a distinctive morphology and memorable common name have given death adders an iconic status within Australian popular culture, the biology of these animals remains poorly known. Most information comes from studies on captive animals [31]–[34]. By far the most detailed field studies on death adders have been based on a single population of *Acanthophis*: northern death adders (*A. praelongus*) near the tropical city of Darwin. Studies on the Darwin population have quantified physiological traits (such as seasonal variation in energy budgets [11]), ecological traits such as movements and demography (based on mark-recapture and radio-telemetry) [12], and behavioral traits such as caudal luring and prey-handling [13]–[15]. The vulnerability of these snakes to an invasive toxic anuran has been examined in detail [16]–[20], as have costs of reproduction [21] and the fitness consequences of modified maternal thermoregulatory regimes during gestation [22]. The only other *Acanthophis* taxon that has been the subject of even superficial ecological studies is an insular population in northern Queensland [23].

The detailed studies on *A. praelongus* have supported several hypotheses about the biology of death adders: for example, they have shown that the caudal lure is effective at attracting some but not all potential prey, with vulnerability greater for some prey taxa [14] and some body sizes [32]. Hence, reliance on this tactic may influence dietary composition [10]. There is, however, less support for Shine's (1980) suggestion that ambush predation is functionally linked to a “slow” life history (i.e. low growth rates, delayed maturation and infrequent reproduction) [10]. Such a link has been supported by studies on other Australian snake species – for example, the ambush predator *Hoplocephalus bungaroides* has a “slower” life history than do sympatric active-foraging elapid species [35], and ambush foragers may be at higher risk of extinction than active-foragers, putatively reflecting those slower life histories [36]. However, studies on the biology of tropical death adders have challenged earlier generalizations based on the southeastern *A. antarcticus*. For example, the tropical *A. praelongus* has rapid growth and early maturation (males at 1 year, females at 2 years) [12], and these snakes are highly mobile rather than sedentary, as might be expected for an ambush forager [22].

The conclusion that death adders encompass a broad range in ecological attributes is supported by the current study. Most obviously, the general body form of these animals varies from extremely thickset (as in *A. praelongus*) to relatively slender (as in *A. wellsi*: see Fig. 1). That diversity does not challenge the strong evolutionary convergence between death adders and other ambush-foraging squamates, because most ambush-foraging squamate lineages encompass equivalent diversity in body shapes. For example, a range in morphotypes from thickset to elongate is evident within ambush-foraging viperids (e.g. *Bitis* to *Lachesis*), pythonids (e.g. *Python curtus* to *Morelia kinghorni*), boids (e.g. *Candoia aspera* to *Corallus hortulanus*) and colubrids (e.g. *Uromacer catesbyi* to *U. frenatus*) [37]. Similarly, the ambush-foraging pygopodid lizard *Lialis burtonis* is more elongate than are any of the *Acanthophis* species [38], and some arboreal snake taxa that rely at least partly on ambush predation are very slender-bodied, perhaps reflecting camouflage on branches rather than in leaf litter e.g. *Boiga irregularis* [39] and *Thelotornis capensis* [40].

Field studies could usefully address relationships between body shapes and foraging tactics within each of these lineages. It is clear that ambush foraging and active searching are the ends of a continuum: individual snakes may use both tactics as a function of local environmental conditions [41]–[43], snake body size (e.g. *Agkistrodon piscivorus*) [44], season (e.g. *Thamnophis cyrtopsis*) [4], and sex (e.g. *Acrochordus arafurae*) [45]. The strong diversity in body shapes within *Acanthophis* (Fig. 1) suggests that they offer an excellent system with which to explore the relative importance of alternative foraging tactics, the environmental and phenotypic traits that influence the use of those tactics, and the consequences of those tactics for prey types and sizes, and for snake feeding rates. Do heavy-bodied taxa within *Acanthophis* rely more on ambush, and slender species more on active foraging? And do any such interspecific or sex-based divergences in foraging tactics translate into divergences in prey choice or activity patterns? Laboratory studies have shown that different prey taxa elicit different caudal-luring responses [46] and prey-consumption methods to deal with postmortem defenses [13], that propensity to lure changes ontogenetically [46], that some caudal lures are sexually dichromatic [47], [48], and that lures are more effective at attracting some potential prey types than others [14]. Snake foraging responses and tactics can be reliably quantified even in small enclosures [46], [49]. Quantitative information on the relationships (if any) between snake morphology, foraging tactics and feeding success could clarify a range of issues, including the question of why such a wide range of morphologies has evolved even within a closely related guild of species that apparently forage in similar ways on similar kinds of prey.

Unsurprisingly, diets vary among death adder species (Table 1, Fig. 5). Much of that variation presumably reflects availability: the spectrum of prey types accessible to a desert adder differs from that encountered by a rainforest species. Shifts in dietary composition with snake body size (Fig. 6A) plausibly reflect the importance of a predator's body size for its ability to capture, subdue and ingest relatively large prey items: a trend for larger snakes to consume larger prey items is widespread but not universal among snakes [50], [51]. Ontogenetic (size-related) shifts in foraging times and places also might contribute to intraspecific and sex-based niche partitioning [44], [52]. A general pattern for lizards to comprise a higher proportion of prey items in male adders than in conspecific, usually larger females (Fig. 6B) is more puzzling. The prey types more often taken by females (mammals and frogs) are active nocturnally, whereas many of the lizards taken by death adders are diurnally active taxa (e.g. *Carlia, Cryptoblepharus, Eulamprus* spp.; Table 1). If these lizards are indeed captured by day, then male death adders may depend more upon diurnal foraging than do females. The reverse shift occurs in ambush-foraging green pythons *Morelia viridis*: they shift from diurnal to nocturnal hunting as they grow larger, but females continue to forage by day whereas males hunt primarily at night [53], thereby consuming fewer diurnal lizards and birds than do female conspecifics [54]. Dietary divergences between the sexes are common in snakes, and often based on sex divergences in body size, in seasonality of feeding, and/or in habitat use or times of activity and timing of reproduction [55], [56]. Head shapes may co-vary with divergence in prey types between sexes [57], [58], and offer a possible explanation for the diversity of patterns in sexually dimorphic head sizes and shapes among death adders. However, our data are not sufficiently extensive to document interspecific variation (if any) in the extent of sex-based dietary divergence. Studies on captive *Acanthophis* could usefully explore the degree to which male and female adders forage by night versus by day; for example, do ambient light levels affect whether or not the approach of a potential prey item elicits caudal luring by male versus female adders?

If all *Acanthophis* species do indeed obtain their prey by ambush predation rather than active searching (as is frequently inferred, but remains poorly documented), the interspecific diversity in many of the traits that we have measured argues against any straightforward impact of foraging mode on those traits. For example, we observed wide variation in population structure (% juveniles, adult sex ratios) of museum samples. That variation might of course reflect collecting biases rather than underlying demography. A general trend for males to dominate in museum collections of large-bodied elapid species likely reflects the greater vulnerability of mate-searching males to collectors [9]; a male snake's single-minded focus on females may render it less reactive to the approach of humans [59]. Such biases are likely to be especially strong in taxa that are well-camouflaged, and rarely move around (like many *Acanthophis*), because an immobile animal may be relatively safe; if dispersal is both rare and dangerous, adult males may be the group especially at risk. Plausibly, local habitat features may affect the degree to which mobility increases exposure to collectors. It is difficult to offer explanations for interspecific diversity in other traits, such as relative head sizes and head shapes, and sexual divergences in these characteristics, without additional information on diets, seasonality, and field ecology.

In contrast to that interspecific diversity, some traits that we measured were similar among taxa. For example, females consistently attained mean adult body sizes about 20% larger than those of adult males (Fig. 2C). That pattern supports the hypothesis that females grow larger than males in snake species in which males do not engage in physical battles for mating opportunities [60]. Male-male combat has never been reported in *Acanthophis* [60]. The relatively longer tails of males than of females also accords with general patterns among snakes [61], [62]. Sexual selection, and the need to accommodate the hemipenes and associated musculature within the tailbase, may favor relatively long tails in males [61]–[63], and the magnitude of sexual disparity in relative tail length tends to be greater in relatively heavy-bodied (and thus, short-tailed) taxa [61]. Given the broad similarity in mean adult body sizes, the similarity in mean litter sizes is unsurprising under the hypothesis that snake reproductive output is maximized relative to maternal abdominal volume [64]. However, that conservatism is inconsistent with life-history models that predict divergent patterns of reproductive output in tropical as compared to temperate-zone or arid-zone species [65].

One clear generality from our data is that death adders feed on a wider variety of prey taxa, and take somewhat different taxa of prey, than do most similar-sized Australian elapids. The majority of elapids that are similar in overall size (body length, body mass and/or head size) to death adders take scincid lizards and/or frogs as their primary prey (e.g. *Cryptophis, Demansia, Hemiaspis*) [8]. Most of the skinks taken by such snakes are small taxa (e.g. *Carlia, Lampropholis*) whereas death adders often take larger skinks (e.g. *Ctenotus, Egernia, Eulamprus*) and agamids (e.g. *Amphibolurus, Ctenophorus, Intellogama*; Table 1). Although birds are relatively rare prey, they were recorded in four of the six *Acanthophis* taxa that we examined, and constituted 3% of all prey records. This is a higher proportion than in any other Australian elapid taxa except the arboreal rough-scaled snakes (*Tropidechis carinatus*, 7%) and the much larger (to>2 m SVL) taipans and king brown snakes (*Oxyuranus scutellatus, Pseudechis australis*, both 5%)[9]. Mammals similarly comprised 21% of prey items in death adders, but are rare prey items in the diets of most Australian elapid snakes (<5% of the diet in all species of all but eight genera) [9]; the exceptions again are ambush predators (*Acanthophis, Echiopsis, Hoplocephalus*), very large species (*Notechis, Oxyuranus, Pseudechis, Pseudonaja*), and the enigmatic *Tropidechis*, whose foraging mode remains unstudied [9], [66]. Thus, although *Acanthophis* resemble other Australian elapids in feeding more often on lizards than on any other prey type, the proportion of the diet comprised of lizards is lower, and the types of lizards consumed are different, than for most other Australian elapid snakes. The similarity in dietary composition between adders and other ambush-foragers such as *Echiopsis* [67] and *Hoplocephalus* [68] supports the idea that ambush foraging provides access to a different (and broader) range of potential prey types than are accessible to an active searching predator. Experimental studies on invertebrate predators have reached the same conclusion [69].

Museum specimens can tell us a great deal, but cannot address many critical dimensions of snake ecology. The putative importance of foraging mode in snake biology identifies the need for field studies (telemetry-based) on snake movements, and laboratory studies on feeding behavior, to fill critical gaps in knowledge. Until we know more about the importance of ambush foraging in the lives of these snakes, we cannot begin to tease apart interspecific, ontogenetic and sex-based variation not only in dietary composition, but also in morphological traits that plausibly are linked to foraging tactics. The morphological and dietary diversity within *Acanthophis* offers great opportunities to clarify questions such as the functional significance of heavyset versus elongate morphologies in snakes, of color polymorphism in ambush predators, and the determinants of ontogenetic and sex-based divergences in diets and morphology. Our dissections of museum specimens tell us that the enormous range of climates and habitats occupied by death adders is accompanied by a substantial level of variation in some traits (such as body shapes and diets), but not others (such as mean adult body lengths, the degree of sexual size dimorphism, or mean litter sizes). An understanding of the adaptive significance of those patterns will require dedicated field studies on these iconic snakes.

## Supporting Information

Table S1 *Acanthophis* museum specimens used in analysis. “Original Species Name” is identification used in museum collection, whereas “Taxon for Analysis” is identification used for this paper. AUS  =  Australia, PNG  =  New Guinea.
(DOCX)
