# Supplementary material for: Morphology, Reproduction and Diet in Australian and Papuan Death Adders (Acanthophis, Elapidae)
Source: PLoS One. 2014 Apr 9;9(4):e94216. doi: 10.1371/journal.pone.0094216 (PMC3981772; doi:10.1371/journal.pone.0094216)
Supplement: Table S1 — Acanthophis museum specimens used in analysis. “Original Species Name” is identification used in museum collection, whereas “Taxon for Analysis” is identification used for this paper. AUS = Australia, PNG = New Guinea. (DOCX) [file pone.0094216.s001.docx]

**Supplementary Material**

**Table S1.** *Acanthophis* museum specimens used in analysis. “Original Species Name” is identification used in

museum collection, whereas “Taxon for Analysis” is identification used for this paper. AUS = Australia, PNG = New Guinea.

| **Original Species Name** | **Taxon for Analysis** | **Museum** | **Catalog Number** |
| --- | --- | --- | --- |
| *A. abdi* | *A. wellsi* | Western Australian Museum | WAM 104188 |
| *A. abdi* | *A. wellsi* | Western Australian Museum | WAM 113114 |
| *A. abdi* | *A. wellsi* | Western Australian Museum | WAM 113166 |
| *A. abdi* | *A. wellsi* | Western Australian Museum | WAM 113167 |
| *A. abdi* | *A. wellsi* | Western Australian Museum | WAM 113374 |
| *A. abdi* | *A. wellsi* | Western Australian Museum | WAM 113377 |
| *A. abdi* | *A. wellsi* | Western Australian Museum | WAM 113378 |
| *A. abdi* | *A. wellsi* | Western Australian Museum | WAM 113432 |
| *A. abdi* | *A. wellsi* | Western Australian Museum | WAM 114901 |
| *A. abdi* | *A. wellsi* | Western Australian Museum | WAM 116934 |
| *A. abdi* | *A. wellsi* | Western Australian Museum | WAM 119365 |
| *A. abdi* | *A. wellsi* | Western Australian Museum | WAM 119366 |
| *A. abdi* | *A. wellsi* | Western Australian Museum | WAM 119367 |
| *A. abdi* | *A. wellsi* | Western Australian Museum | WAM 12280 |
| *A. abdi* | *A. wellsi* | Western Australian Museum | WAM 12672 |
| *A. abdi* | *A. wellsi* | Western Australian Museum | WAM 20239 |
| *A. abdi* | *A. wellsi* | Western Australian Museum | WAM 21538 |
| *A. abdi* | *A. wellsi* | Western Australian Museum | WAM 26759 |
| *A. abdi* | *A. wellsi* | Western Australian Museum | WAM 26822 |
| *A. abdi* | *A. wellsi* | Western Australian Museum | WAM 46836 |
| *A. abdi* | *A. wellsi* | Western Australian Museum | WAM 61357 |
| *A. abdi* | *A. wellsi* | Western Australian Museum | WAM 61495 |
| *A. abdi* | *A. wellsi* | Western Australian Museum | WAM 67921 |
| *A. abdi* | *A. wellsi* | Western Australian Museum | WAM 71228 |
| *A. abdi* | *A. wellsi* | Western Australian Museum | WAM 73731 |
| *A. abdi* | *A. wellsi* | Western Australian Museum | WAM 75003 |
| *A. antarcticus* | *A. antarcticus* PNG | Australian Museum | AM 3383 |
| *A. antarcticus* | *A. antarcticus* PNG | Australian Museum | AM 3384 |
| *A. antarcticus* | *A. antarcticus* PNG | Australian Museum | AM 8881 |
| *A. antarcticus* | *A. antarcticus* PNG | Australian Museum | AM 8881 |
| *A. antarcticus* | *A. antarcticus* PNG | Australian Museum | AM 11829 |
| *A. antarcticus* | *A. antarcticus* PNG | Australian Museum | AM 12226 |
| *A. antarcticus* | *A. antarcticus* PNG | Australian Museum | AM 12975 |
| *A. antarcticus* | *A. antarcticus* PNG | Australian Museum | AM 13705 |
| *A. antarcticus* | *A. antarcticus* PNG | Australian Museum | AM 13825 |
| *A. antarcticus* | *A. antarcticus* PNG | Australian Museum | AM 13826 |
| *A. antarcticus* | *A. antarcticus* PNG | Australian Museum | AM 14352 |
| *A. antarcticus* | *A. antarcticus* PNG | Australian Museum | AM 14353 |
| *A. antarcticus* | *A. antarcticus* PNG | Australian Museum | AM 14423 |
| *A. antarcticus* | *A. antarcticus* PNG | Australian Museum | AM 14423 |
| *A. antarcticus* | *A. antarcticus* PNG | Australian Museum | AM 14480 |
| *A. antarcticus* | *A. antarcticus* PNG | Australian Museum | AM 14779 |
| *A. antarcticus* | *A. antarcticus* PNG | Australian Museum | AM 14851 |
| *A. antarcticus* | *A. antarcticus* PNG | Australian Museum | AM 14852 |
| *A. antarcticus* | *A. antarcticus* PNG | Australian Museum | AM 15750 |
| *A. antarcticus* | *A. antarcticus* PNG | Australian Museum | AM 15949 |
| *A. antarcticus* | *A. antarcticus* PNG | Australian Museum | AM 23953 |
| *A. antarcticus* | *A. antarcticus* PNG | Australian Museum | AM 23953 |
| *A. antarcticus* | *A. antarcticus* PNG | Australian Museum | AM 23960 |
| *A. antarcticus* | *A. antarcticus* PNG | Australian Museum | AM 23961 |
| *A. antarcticus* | *A. antarcticus* PNG | Australian Museum | AM 23962 |
| *A. antarcticus* | *A. antarcticus* PNG | Australian Museum | AM 25368 |
| *A. antarcticus* | *A. antarcticus* PNG | Australian Museum | AM 66760 |
| *A. antarcticus* | *A. antarcticus* PNG | Australian Museum | AM 66772 |
| *A. antarcticus* | *A. antarcticus* PNG | Australian Museum | AM 69980 |
| *A. antarcticus* | *A. antarcticus* PNG | Bishop Museum | BPBM 2029 |
| *A. antarcticus* | *A. antarcticus* PNG | Bishop Museum | BPBM 2776 |
| *A. antarcticus* | *A. antarcticus* PNG | Bishop Museum | BPBM 3653 |
| *A. antarcticus* | *A. antarcticus* PNG | Bishop Museum | BPBM 3776 |
| *A. antarcticus* | *A. antarcticus* PNG | Bishop Museum | BPBM 3851 |
| *A. antarcticus* | *A. antarcticus* PNG | Bishop Museum | BPBM 5028 |
| *A. antarcticus* | *A. antarcticus* PNG | Bishop Museum | BPBM 5717 |
| *A. antarcticus* | *A. antarcticus* PNG | California Academy of Sciences | CAS 12970 |
| *A. antarcticus* | *A. antarcticus* PNG | California Academy of Sciences | CAS 12971 |
| *A. antarcticus* | *A. antarcticus* PNG | California Academy of Sciences | CAS 13229 |
| *A. antarcticus* | *A. antarcticus* PNG | California Academy of Sciences | CAS 13237 |
| *A. antarcticus* | *A. antarcticus* PNG | California Academy of Sciences | CAS 13238 |
| *A. antarcticus* | *A. antarcticus* PNG | California Academy of Sciences | CAS 53987 |
| *A. antarcticus* | *A. antarcticus* PNG | California Academy of Sciences | CAS 98662 |
| *A. antarcticus* | *A. antarcticus* PNG | California Academy of Sciences | CAS 109908 |
| *A. antarcticus* | *A. antarcticus* PNG | California Academy of Sciences | CAS 109909 |
| *A. antarcticus* | *A. antarcticus* PNG | California Academy of Sciences | CAS 109910 |
| *A. antarcticus* | *A. antarcticus* PNG | California Academy of Sciences | CAS 109911 |
| *A. antarcticus* | *A. antarcticus* PNG | California Academy of Sciences | CAS 109912 |
| *A. antarcticus* | *A. antarcticus* PNG | California Academy of Sciences | CAS 109913 |
| *A. antarcticus* | *A. antarcticus* PNG | California Academy of Sciences | CAS 109914 |
| *A. antarcticus* | *A. antarcticus* PNG | California Academy of Sciences | CAS 109915 |
| *A. antarcticus* | *A. antarcticus* PNG | California Academy of Sciences | CAS 109916 |
| *A. antarcticus* | *A. antarcticus* PNG | California Academy of Sciences | CAS 109917 |
| *A. antarcticus* | *A. antarcticus* PNG | California Academy of Sciences | CAS 109918 |
| *A. antarcticus* | *A. antarcticus* PNG | California Academy of Sciences | CAS 110383 |
| *A. antarcticus* | *A. antarcticus* PNG | California Academy of Sciences | CAS 110384 |
| *A. antarcticus* | *A. antarcticus* PNG | California Academy of Sciences | CAS 110385 |
| *A. antarcticus* | *A. antarcticus* PNG | California Academy of Sciences | CAS 110386 |
| *A. antarcticus* | *A. antarcticus* PNG | California Academy of Sciences | CAS 110387 |
| *A. antarcticus* | *A. antarcticus* PNG | California Academy of Sciences | CAS 110388 |
| *A. antarcticus* | *A. antarcticus* PNG | California Academy of Sciences | CAS 113632 |
| *A. antarcticus* | *A. antarcticus* PNG | California Academy of Sciences | CAS 113633 |
| *A. antarcticus* | *A. antarcticus* PNG | California Academy of Sciences | CAS 113676 |
| *A. antarcticus* | *A. antarcticus* PNG | California Academy of Sciences | CAS 113677 |
| *A. antarcticus* | *A. antarcticus* PNG | California Academy of Sciences | CAS 113678 |
| *A. antarcticus* | *A. antarcticus* PNG | California Academy of Sciences | CAS 113679 |
| *A. antarcticus* | *A. antarcticus* PNG | California Academy of Sciences | CAS 113680 |
| *A. antarcticus* | *A. antarcticus* PNG | California Academy of Sciences | CAS 113681 |
| *A. antarcticus* | *A. antarcticus* PNG | California Academy of Sciences | CAS 113682 |
| *A. antarcticus* | *A. antarcticus* PNG | California Academy of Sciences | CAS 113683 |
| *A. antarcticus* | *A. antarcticus* PNG | California Academy of Sciences | CAS 113684 |
| *A. antarcticus* | *A. antarcticus* PNG | California Academy of Sciences | CAS 113685 |
| *A. antarcticus* | *A. antarcticus* PNG | California Academy of Sciences | CAS 113686 |
| *A. antarcticus* | *A. antarcticus* PNG | California Academy of Sciences | CAS 113687 |
| *A. antarcticus* | *A. antarcticus* PNG | California Academy of Sciences | CAS 113688 |
| *A. antarcticus* | *A. antarcticus* PNG | California Academy of Sciences | CAS 113689 |
| *A. antarcticus* | *A. antarcticus* PNG | California Academy of Sciences | CAS 113690 |
| *A. antarcticus* | *A. antarcticus* PNG | California Academy of Sciences | CAS 113691 |
| *A. antarcticus* | *A. antarcticus* PNG | California Academy of Sciences | CAS 113692 |
| *A. antarcticus* | *A. antarcticus* PNG | California Academy of Sciences | CAS 113693 |
| *A. antarcticus* | *A. antarcticus* PNG | California Academy of Sciences | CAS 113694 |
| *A. antarcticus* | *A. antarcticus* PNG | California Academy of Sciences | CAS 113695 |
| *A. antarcticus* | *A. antarcticus* PNG | California Academy of Sciences | CAS 113696 |
| *A. antarcticus* | *A. antarcticus* PNG | California Academy of Sciences | CAS 113697 |
| *A. antarcticus* | *A. antarcticus* PNG | California Academy of Sciences | CAS 113698 |
| *A. antarcticus* | *A. antarcticus* PNG | California Academy of Sciences | CAS 113699 |
| *A. antarcticus* | *A. antarcticus* PNG | California Academy of Sciences | CAS 113700 |
| *A. antarcticus* | *A. antarcticus* PNG | California Academy of Sciences | CAS 113701 |
| *A. antarcticus* | *A. antarcticus* PNG | California Academy of Sciences | CAS 113702 |
| *A. antarcticus* | *A. antarcticus* PNG | California Academy of Sciences | CAS 113703 |
| *A. antarcticus* | *A. antarcticus* PNG | California Academy of Sciences | CAS 113704 |
| *A. antarcticus* | *A. antarcticus* PNG | California Academy of Sciences | CAS 113705 |
| *A. antarcticus* | *A. antarcticus* PNG | California Academy of Sciences | CAS 113706 |
| *A. antarcticus* | *A. antarcticus* PNG | California Academy of Sciences | CAS 113707 |
| *A. antarcticus* | *A. antarcticus* PNG | California Academy of Sciences | CAS 113708 |
| *A. antarcticus* | *A. antarcticus* PNG | California Academy of Sciences | CAS 113709 |
| *A. antarcticus* | *A. antarcticus* PNG | California Academy of Sciences | CAS 114115 |
| *A. antarcticus* | *A. antarcticus* PNG | California Academy of Sciences | CAS 114116 |
| *A. antarcticus* | *A. antarcticus* PNG | California Academy of Sciences | CAS 114117 |
| *A. antarcticus* | *A. antarcticus* PNG | California Academy of Sciences | CAS 114118 |
| *A. antarcticus* | *A. antarcticus* PNG | California Academy of Sciences | CAS 114119 |
| *A. antarcticus* | *A. antarcticus* PNG | California Academy of Sciences | CAS 114120 |
| *A. antarcticus* | *A. antarcticus* PNG | California Academy of Sciences | CAS 114121 |
| *A. antarcticus* | *A. antarcticus* PNG | California Academy of Sciences | CAS 114122 |
| *A. antarcticus* | *A. antarcticus* PNG | California Academy of Sciences | CAS 114123 |
| *A. antarcticus* | *A. antarcticus* PNG | California Academy of Sciences | CAS 114124 |
| *A. antarcticus* | *A. antarcticus* PNG | California Academy of Sciences | CAS 114125 |
| *A. antarcticus* | *A. antarcticus* PNG | California Academy of Sciences | CAS 114126 |
| *A. antarcticus* | *A. antarcticus* PNG | California Academy of Sciences | CAS 114127 |
| *A. antarcticus* | *A. antarcticus* PNG | California Academy of Sciences | CAS 114128 |
| *A. antarcticus* | *A. antarcticus* PNG | California Academy of Sciences | CAS 114129 |
| *A. antarcticus* | *A. antarcticus* PNG | California Academy of Sciences | CAS 114130 |
| *A. antarcticus* | *A. antarcticus* PNG | California Academy of Sciences | CAS 114131 |
| *A. antarcticus* | *A. antarcticus* PNG | California Academy of Sciences | CAS 114132 |
| *A. antarcticus* | *A. antarcticus* PNG | California Academy of Sciences | CAS 114133 |
| *A. antarcticus* | *A. antarcticus* PNG | California Academy of Sciences | CAS 114134 |
| *A. antarcticus* | *A. antarcticus* PNG | California Academy of Sciences | CAS 114135 |
| *A. antarcticus* | *A. antarcticus* PNG | California Academy of Sciences | CAS 114136 |
| *A. antarcticus* | *A. antarcticus* PNG | California Academy of Sciences | CAS 114137 |
| *A. antarcticus* | *A. antarcticus* PNG | California Academy of Sciences | CAS 114138 |
| *A. antarcticus* | *A. antarcticus* PNG | California Academy of Sciences | CAS 114139 |
| *A. antarcticus* | *A. antarcticus* PNG | California Academy of Sciences | CAS 114140 |
| *A. antarcticus* | *A. antarcticus* PNG | California Academy of Sciences | CAS 114141 |
| *A. antarcticus* | *A. antarcticus* PNG | California Academy of Sciences | CAS 114142 |
| *A. antarcticus* | *A. antarcticus* PNG | California Academy of Sciences | CAS 114143 |
| *A. antarcticus* | *A. antarcticus* PNG | California Academy of Sciences | CAS 114144 |
| *A. antarcticus* | *A. antarcticus* PNG | California Academy of Sciences | CAS 114145 |
| *A. antarcticus* | *A. antarcticus* PNG | California Academy of Sciences | CAS 114146 |
| *A. antarcticus* | *A. antarcticus* PNG | California Academy of Sciences | CAS 114147 |
| *A. antarcticus* | *A. antarcticus* PNG | California Academy of Sciences | CAS 114148 |
| *A. antarcticus* | *A. antarcticus* PNG | California Academy of Sciences | CAS 114149 |
| *A. antarcticus* | *A. antarcticus* PNG | California Academy of Sciences | CAS 114150 |
| *A. antarcticus* | *A. antarcticus* PNG | California Academy of Sciences | CAS 114151 |
| *A. antarcticus* | *A. antarcticus* PNG | California Academy of Sciences | CAS 114152 |
| *A. antarcticus* | *A. antarcticus* PNG | California Academy of Sciences | CAS 114153 |
| *A. antarcticus* | *A. antarcticus* PNG | California Academy of Sciences | CAS 114154 |
| *A. antarcticus* | *A. antarcticus* PNG | California Academy of Sciences | CAS 114155 |
| *A. antarcticus* | *A. antarcticus* PNG | California Academy of Sciences | CAS 114156 |
| *A. antarcticus* | *A. antarcticus* PNG | California Academy of Sciences | CAS 114157 |
| *A. antarcticus* | *A. antarcticus* PNG | California Academy of Sciences | CAS 114158 |
| *A. antarcticus* | *A. antarcticus* PNG | California Academy of Sciences | CAS 114159 |
| *A. antarcticus* | *A. antarcticus* PNG | California Academy of Sciences | CAS 114160 |
| *A. antarcticus* | *A. antarcticus* PNG | California Academy of Sciences | CAS 114161 |
| *A. antarcticus* | *A. antarcticus* PNG | California Academy of Sciences | CAS 114162 |
| *A. antarcticus* | *A. antarcticus* PNG | California Academy of Sciences | CAS 114163 |
| *A. antarcticus* | *A. antarcticus* PNG | California Academy of Sciences | CAS 114164 |
| *A. antarcticus* | *A. antarcticus* PNG | California Academy of Sciences | CAS 114165 |
| *A. antarcticus* | *A. antarcticus* PNG | California Academy of Sciences | CAS 114166 |
| *A. antarcticus* | *A. antarcticus* PNG | California Academy of Sciences | CAS 114167 |
| *A. antarcticus* | *A. antarcticus* PNG | California Academy of Sciences | CAS 114168 |
| *A. antarcticus* | *A. antarcticus* PNG | California Academy of Sciences | CAS 114169 |
| *A. antarcticus* | *A. antarcticus* PNG | California Academy of Sciences | CAS 114170 |
| *A. antarcticus* | *A. antarcticus* PNG | California Academy of Sciences | CAS 114171 |
| *A. antarcticus* | *A. antarcticus* PNG | California Academy of Sciences | CAS 114172 |
| *A. antarcticus* | *A. antarcticus* PNG | California Academy of Sciences | CAS 114173 |
| *A. antarcticus* | *A. antarcticus* PNG | California Academy of Sciences | CAS 114174 |
| *A. antarcticus* | *A. antarcticus* PNG | California Academy of Sciences | CAS 114175 |
| *A. antarcticus* | *A. antarcticus* PNG | California Academy of Sciences | CAS 114176 |
| *A. antarcticus* | *A. antarcticus* PNG | California Academy of Sciences | CAS 114177 |
| *A. antarcticus* | *A. antarcticus* PNG | California Academy of Sciences | CAS 114178 |
| *A. antarcticus* | *A. antarcticus* PNG | California Academy of Sciences | CAS 114179 |
| *A. antarcticus* | *A. antarcticus* PNG | California Academy of Sciences | CAS 114180 |
| *A. antarcticus* | *A. antarcticus* PNG | California Academy of Sciences | CAS 114181 |
| *A. antarcticus* | *A. antarcticus* PNG | California Academy of Sciences | CAS 114182 |
| *A. antarcticus* | *A. antarcticus* PNG | California Academy of Sciences | CAS 114183 |
| *A. antarcticus* | *A. antarcticus* PNG | California Academy of Sciences | CAS 114184 |
| *A. antarcticus* | *A. antarcticus* PNG | California Academy of Sciences | CAS 114185 |
| *A. antarcticus* | *A. antarcticus* PNG | California Academy of Sciences | CAS 114186 |
| *A. antarcticus* | *A. antarcticus* PNG | California Academy of Sciences | CAS 114187 |
| *A. antarcticus* | *A. antarcticus* PNG | California Academy of Sciences | CAS 114188 |
| *A. antarcticus* | *A. antarcticus* PNG | California Academy of Sciences | CAS 114189 |
| *A. antarcticus* | *A. antarcticus* PNG | California Academy of Sciences | CAS 114190 |
| *A. antarcticus* | *A. antarcticus* PNG | California Academy of Sciences | CAS 114191 |
| *A. antarcticus* | *A. antarcticus* PNG | California Academy of Sciences | CAS 114192 |
| *A. antarcticus* | *A. antarcticus* PNG | California Academy of Sciences | CAS 114193 |
| *A. antarcticus* | *A. antarcticus* PNG | California Academy of Sciences | CAS 114194 |
| *A. antarcticus* | *A. antarcticus* PNG | California Academy of Sciences | CAS 114195 |
| *A. antarcticus* | *A. antarcticus* PNG | California Academy of Sciences | CAS 114196 |
| *A. antarcticus* | *A. antarcticus* PNG | California Academy of Sciences | CAS 114197 |
| *A. antarcticus* | *A. antarcticus* PNG | California Academy of Sciences | CAS 114198 |
| *A. antarcticus* | *A. antarcticus* PNG | California Academy of Sciences | CAS 114199 |
| *A. antarcticus* | *A. antarcticus* PNG | California Academy of Sciences | CAS 114200 |
| *A. antarcticus* | *A. antarcticus* PNG | California Academy of Sciences | CAS 114201 |
| *A. antarcticus* | *A. antarcticus* PNG | California Academy of Sciences | CAS 114202 |
| *A. antarcticus* | *A. antarcticus* PNG | California Academy of Sciences | CAS 114203 |
| *A. antarcticus* | *A. antarcticus* PNG | California Academy of Sciences | CAS 114204 |
| *A. antarcticus* | *A. antarcticus* PNG | California Academy of Sciences | CAS 114205 |
| *A. antarcticus* | *A. antarcticus* PNG | California Academy of Sciences | CAS 114206 |
| *A. antarcticus* | *A. antarcticus* PNG | California Academy of Sciences | CAS 114207 |
| *A. antarcticus* | *A. antarcticus* PNG | California Academy of Sciences | CAS 114208 |
| *A. antarcticus* | *A. antarcticus* PNG | California Academy of Sciences | CAS 118883 |
| *A. antarcticus* | *A. antarcticus* PNG | California Academy of Sciences | CAS 118885 |
| *A. antarcticus* | *A. antarcticus* PNG | California Academy of Sciences | CAS 118890 |
| *A. antarcticus* | *A. antarcticus* PNG | California Academy of Sciences | CAS 118891 |
| *A. antarcticus* | *A. antarcticus* PNG | California Academy of Sciences | CAS 118892 |
| *A. antarcticus* | *A. antarcticus* PNG | California Academy of Sciences | CAS 118893 |
| *A. antarcticus* | *A. antarcticus* PNG | California Academy of Sciences | CAS 118894 |
| *A. antarcticus* | *A. antarcticus* PNG | California Academy of Sciences | CAS 118895 |
| *A. antarcticus* | *A. antarcticus* PNG | California Academy of Sciences | CAS 118896 |
| *A. antarcticus* | *A. antarcticus* PNG | California Academy of Sciences | CAS 118897 |
| *A. antarcticus* | *A. antarcticus* PNG | California Academy of Sciences | CAS 118898 |
| *A. antarcticus* | *A. antarcticus* PNG | California Academy of Sciences | CAS 118899 |
| *A. antarcticus* | *A. antarcticus* PNG | California Academy of Sciences | CAS 118900 |
| *A. antarcticus* | *A. antarcticus* PNG | California Academy of Sciences | CAS 118901 |
| *A. antarcticus* | *A. antarcticus* PNG | California Academy of Sciences | CAS 118902 |
| *A. antarcticus* | *A. antarcticus* PNG | California Academy of Sciences | CAS 118903 |
| *A. antarcticus* | *A. antarcticus* PNG | California Academy of Sciences | CAS 118904 |
| *A. antarcticus* | *A. antarcticus* PNG | California Academy of Sciences | CAS 118918 |
| *A. antarcticus* | *A. antarcticus* PNG | California Academy of Sciences | CAS 118919 |
| *A. antarcticus* | *A. antarcticus* PNG | California Academy of Sciences | CAS 118920 |
| *A. antarcticus* | *A. antarcticus* PNG | California Academy of Sciences | CAS 118921 |
| *A. antarcticus* | *A. antarcticus* PNG | California Academy of Sciences | CAS 118922 |
| *A. antarcticus* | *A. antarcticus* PNG | California Academy of Sciences | CAS 118923 |
| *A. antarcticus* | *A. antarcticus* PNG | California Academy of Sciences | CAS 118924 |
| *A. antarcticus* | *A. antarcticus* PNG | California Academy of Sciences | CAS 118925 |
| *A. antarcticus* | *A. antarcticus* PNG | California Academy of Sciences | CAS 118926 |
| *A. antarcticus* | *A. antarcticus* PNG | California Academy of Sciences | CAS 118927 |
| *A. antarcticus* | *A. antarcticus* PNG | California Academy of Sciences | CAS 118928 |
| *A. antarcticus* | *A. antarcticus* PNG | California Academy of Sciences | CAS 118929 |
| *A. antarcticus* | *A. antarcticus* PNG | California Academy of Sciences | CAS 118930 |
| *A. antarcticus* | *A. antarcticus* PNG | California Academy of Sciences | CAS 118931 |
| *A. antarcticus* | *A. antarcticus* PNG | California Academy of Sciences | CAS 118932 |
| *A. antarcticus* | *A. antarcticus* PNG | California Academy of Sciences | CAS 118933 |
| *A. antarcticus* | *A. antarcticus* PNG | California Academy of Sciences | CAS 118934 |
| *A. antarcticus* | *A. antarcticus* PNG | California Academy of Sciences | CAS 118935 |
| *A. antarcticus* | *A. antarcticus* PNG | California Academy of Sciences | CAS 118936 |
| *A. antarcticus* | *A. antarcticus* PNG | California Academy of Sciences | CAS 118937 |
| *A. antarcticus* | *A. antarcticus* PNG | California Academy of Sciences | CAS 118938 |
| *A. antarcticus* | *A. antarcticus* PNG | California Academy of Sciences | CAS 118942 |
| *A. antarcticus* | *A. antarcticus* PNG | California Academy of Sciences | CAS 121225 |
| *A. antarcticus* | *A. antarcticus* PNG | California Academy of Sciences | CAS 127372 |
| *A. antarcticus* | *A. antarcticus* PNG | California Academy of Sciences | CAS 127373 |
| *A. antarcticus* | *A. antarcticus* PNG | California Academy of Sciences | CAS 132235 |
| *A. antarcticus* | *A. antarcticus* PNG | California Academy of Sciences | CAS 132238 |
| *A. antarcticus* | *A. antarcticus* PNG | California Academy of Sciences | CAS 132239 |
| *A. antarcticus* | *A. antarcticus* PNG | California Academy of Sciences | CAS 132240 |
| *A. antarcticus* | *A. antarcticus* PNG | California Academy of Sciences | CAS 132241 |
| *A. antarcticus* | *A. antarcticus* PNG | California Academy of Sciences | CAS 132242 |
| *A. antarcticus* | *A. antarcticus* PNG | California Academy of Sciences | CAS 135227 |
| *A. antarcticus* | *A. antarcticus* PNG | California Academy of Sciences | CAS 135507 |
| *A. antarcticus* | *A. antarcticus* PNG | California Academy of Sciences | CAS 135508 |
| *A. antarcticus* | *A. antarcticus* PNG | California Academy of Sciences | CAS 135509 |
| *A. antarcticus* | *A. antarcticus* PNG | California Academy of Sciences | CAS 135510 |
| *A. antarcticus* | *A. antarcticus* PNG | California Academy of Sciences | CAS 135511 |
| *A. antarcticus* | *A. antarcticus* PNG | California Academy of Sciences | CAS-SU 20903 |
| *A. antarcticus* | *A. antarcticus* PNG | California Academy of Sciences | CAS-SU 20904 |
| *A. antarcticus* | *A. antarcticus* PNG | California Academy of Sciences | CAS-SU 20905 |
| *A. antarcticus* | *A. antarcticus* PNG | California Academy of Sciences | CAS-SU 20906 |
| *A. antarcticus* | *A. antarcticus* PNG | California Academy of Sciences | CAS-SU 20907 |
| *A. antarcticus* | *A. antarcticus* PNG | Carnegie Museum of Natural History | CM 58780 |
| *A. antarcticus* | *A. antarcticus* PNG | Carnegie Museum of Natural History | CM 124131 |
| *A. antarcticus* | *A. antarcticus* PNG | Field Museum of Natural History | FMNH 13955 |
| *A. antarcticus* | *A. antarcticus* PNG | Field Museum of Natural History | FMNH 21734 |
| *A. antarcticus* | *A. antarcticus* PNG | University of Michigan Museum of Zoology | UMMZ 144835 |
| *A. antarcticus* | *A. antarcticus* PNG | University of Michigan Museum of Zoology | UMMZ 210535 |
| *A. antarcticus* | *A. antarcticus* PNG | United States National Museum | USNM 119192 |
| *A. antarcticus* | *A. antarcticus* PNG | United States National Museum | USNM 119516 |
| *A. antarcticus* | *A. antarcticus* PNG | United States National Museum | USNM 166233 |
| *A. antarcticus* | *A. antarcticus* PNG | United States National Museum | USNM 166234 |
| *A. antarcticus* | *A. antarcticus* PNG | United States National Museum | USNM 166235 |
| *A. antarcticus* | *A. antarcticus* PNG | United States National Museum | USNM 166236 |
| *A. antarcticus* | *A. antarcticus* PNG | United States National Museum | USNM 195581 |
| *A. antarcticus* | *A. antarcticus* PNG | United States National Museum | USNM 195582 |
| *A. antarcticus* | *A. antarcticus* PNG | United States National Museum | USNM 195583 |
| *A. antarcticus* | *A. antarcticus* PNG | United States National Museum | USNM 213460 |
| *A. antarcticus* | *A. antarcticus* PNG | United States National Museum | USNM 213461 |
| *A. antarcticus* | *A. antarcticus* PNG | United States National Museum | USNM 213462 |
| *A. antarcticus* | *A. antarcticus* PNG | United States National Museum | USNM 213463 |
| *A. antarcticus* | *A. antarcticus* PNG | United States National Museum | USNM 213464 |
| *A. antarcticus* | *A. antarcticus* PNG | United States National Museum | USNM 237694 |
| *A. antarcticus* | *A. antarcticus* PNG | United States National Museum | USNM 527839 |
| *A. antarcticus* | *A. antarcticus* PNG | United States National Museum | USNM 527840 |
| *A. antarcticus* | *A. antarcticus* PNG | United States National Museum | USNM 527841 |
| *A. antarcticus* | *A. antarcticus* PNG | United States National Museum | USNM 527842 |
| *A. antarcticus* | *A. antarcticus* PNG | University of Texas at Arlington | UTA-R 39077 |
| *A. antarcticus* | *A. antarcticus* southeastern AUS | Australian Museum | AM 440 |
| *A. antarcticus* | *A. antarcticus* southeastern AUS | Australian Museum | AM 440 |
| *A. antarcticus* | *A. antarcticus* southeastern AUS | Australian Museum | AM 587 |
| *A. antarcticus* | *A. antarcticus* southeastern AUS | Australian Museum | AM 1278 |
| *A. antarcticus* | *A. antarcticus* southeastern AUS | Australian Museum | AM 1834 |
| *A. antarcticus* | *A. antarcticus* southeastern AUS | Australian Museum | AM 2317 |
| *A. antarcticus* | *A. antarcticus* southeastern AUS | Australian Museum | AM 2601 |
| *A. antarcticus* | *A. antarcticus* southeastern AUS | Australian Museum | AM 2758 |
| *A. antarcticus* | *A. antarcticus* southeastern AUS | Australian Museum | AM 3290 |
| *A. antarcticus* | *A. antarcticus* southeastern AUS | Australian Museum | AM 3423 |
| *A. antarcticus* | *A. antarcticus* southeastern AUS | Australian Museum | AM 3432 |
| *A. antarcticus* | *A. antarcticus* southeastern AUS | Australian Museum | AM 3432 |
| *A. antarcticus* | *A. antarcticus* southeastern AUS | Australian Museum | AM 3697 |
| *A. antarcticus* | *A. antarcticus* southeastern AUS | Australian Museum | AM 3858 |
| *A. antarcticus* | *A. antarcticus* southeastern AUS | Australian Museum | AM 3881 |
| *A. antarcticus* | *A. antarcticus* southeastern AUS | Australian Museum | AM 4489 |
| *A. antarcticus* | *A. antarcticus* southeastern AUS | Australian Museum | AM 4621 |
| *A. antarcticus* | *A. antarcticus* southeastern AUS | Australian Museum | AM 5764 |
| *A. antarcticus* | *A. antarcticus* southeastern AUS | Australian Museum | AM 5767 |
| *A. antarcticus* | *A. antarcticus* southeastern AUS | Australian Museum | AM 5770 |
| *A. antarcticus* | *A. antarcticus* southeastern AUS | Australian Museum | AM 5770 |
| *A. antarcticus* | *A. antarcticus* southeastern AUS | Australian Museum | AM 5772 |
| *A. antarcticus* | *A. antarcticus* southeastern AUS | Australian Museum | AM 5773 |
| *A. antarcticus* | *A. antarcticus* southeastern AUS | Australian Museum | AM 5776 |
| *A. antarcticus* | *A. antarcticus* southeastern AUS | Australian Museum | AM 5777 |
| *A. antarcticus* | *A. antarcticus* southeastern AUS | Australian Museum | AM 5779 |
| *A. antarcticus* | *A. antarcticus* southeastern AUS | Australian Museum | AM 5793 |
| *A. antarcticus* | *A. antarcticus* southeastern AUS | Australian Museum | AM 6686 |
| *A. antarcticus* | *A. antarcticus* southeastern AUS | Australian Museum | AM 6689 |
| *A. antarcticus* | *A. antarcticus* southeastern AUS | Australian Museum | AM 6690 |
| *A. antarcticus* | *A. antarcticus* southeastern AUS | Australian Museum | AM 6692 |
| *A. antarcticus* | *A. antarcticus* southeastern AUS | Australian Museum | AM 6721 |
| *A. antarcticus* | *A. antarcticus* southeastern AUS | Australian Museum | AM 6779 |
| *A. antarcticus* | *A. antarcticus* southeastern AUS | Australian Museum | AM 7429 |
| *A. antarcticus* | *A. antarcticus* southeastern AUS | Australian Museum | AM 7429 |
| *A. antarcticus* | *A. antarcticus* southeastern AUS | Australian Museum | AM 8084 |
| *A. antarcticus* | *A. antarcticus* southeastern AUS | Australian Museum | AM 8285 |
| *A. antarcticus* | *A. antarcticus* southeastern AUS | Australian Museum | AM 8698 |
| *A. antarcticus* | *A. antarcticus* southeastern AUS | Australian Museum | AM 8698 |
| *A. antarcticus* | *A. antarcticus* southeastern AUS | Australian Museum | AM 8926 |
| *A. antarcticus* | *A. antarcticus* southeastern AUS | Australian Museum | AM 8926 |
| *A. antarcticus* | *A. antarcticus* southeastern AUS | Australian Museum | AM 9445 |
| *A. antarcticus* | *A. antarcticus* southeastern AUS | Australian Museum | AM 9445 |
| *A. antarcticus* | *A. antarcticus* southeastern AUS | Australian Museum | AM 10129 |
| *A. antarcticus* | *A. antarcticus* southeastern AUS | Australian Museum | AM 10130 |
| *A. antarcticus* | *A. antarcticus* southeastern AUS | Australian Museum | AM 10130 |
| *A. antarcticus* | *A. antarcticus* southeastern AUS | Australian Museum | AM 10131 |
| *A. antarcticus* | *A. antarcticus* southeastern AUS | Australian Museum | AM 10132 |
| *A. antarcticus* | *A. antarcticus* southeastern AUS | Australian Museum | AM 10133 |
| *A. antarcticus* | *A. antarcticus* southeastern AUS | Australian Museum | AM 10134 |
| *A. antarcticus* | *A. antarcticus* southeastern AUS | Australian Museum | AM 10135 |
| *A. antarcticus* | *A. antarcticus* southeastern AUS | Australian Museum | AM 10149 |
| *A. antarcticus* | *A. antarcticus* southeastern AUS | Australian Museum | AM 10149 |
| *A. antarcticus* | *A. antarcticus* southeastern AUS | Australian Museum | AM 10150 |
| *A. antarcticus* | *A. antarcticus* southeastern AUS | Australian Museum | AM 10151 |
| *A. antarcticus* | *A. antarcticus* southeastern AUS | Australian Museum | AM 10273 |
| *A. antarcticus* | *A. antarcticus* southeastern AUS | Australian Museum | AM 10421 |
| *A. antarcticus* | *A. antarcticus* southeastern AUS | Australian Museum | AM 11198 |
| *A. antarcticus* | *A. antarcticus* southeastern AUS | Australian Museum | AM 11368 |
| *A. antarcticus* | *A. antarcticus* southeastern AUS | Australian Museum | AM 11527 |
| *A. antarcticus* | *A. antarcticus* southeastern AUS | Australian Museum | AM 11641 |
| *A. antarcticus* | *A. antarcticus* southeastern AUS | Australian Museum | AM 11642 |
| *A. antarcticus* | *A. antarcticus* southeastern AUS | Australian Museum | AM 11696 |
| *A. antarcticus* | *A. antarcticus* southeastern AUS | Australian Museum | AM 11712 |
| *A. antarcticus* | *A. antarcticus* southeastern AUS | Australian Museum | AM 11747 |
| *A. antarcticus* | *A. antarcticus* southeastern AUS | Australian Museum | AM 12032 |
| *A. antarcticus* | *A. antarcticus* southeastern AUS | Australian Museum | AM 12249 |
| *A. antarcticus* | *A. antarcticus* southeastern AUS | Australian Museum | AM 12523 |
| *A. antarcticus* | *A. antarcticus* southeastern AUS | Australian Museum | AM 12933 |
| *A. antarcticus* | *A. antarcticus* southeastern AUS | Australian Museum | AM 12976 |
| *A. antarcticus* | *A. antarcticus* southeastern AUS | Australian Museum | AM 13401 |
| *A. antarcticus* | *A. antarcticus* southeastern AUS | Australian Museum | AM 14799 |
| *A. antarcticus* | *A. antarcticus* southeastern AUS | Australian Museum | AM 15626 |
| *A. antarcticus* | *A. antarcticus* southeastern AUS | Australian Museum | AM 30350 |
| *A. antarcticus* | *A. antarcticus* southeastern AUS | Australian Museum | AM 37417 |
| *A. antarcticus* | *A. antarcticus* southeastern AUS | Australian Museum | AM 41642 |
| *A. antarcticus* | *A. antarcticus* southeastern AUS | Australian Museum | AM 43544 |
| *A. antarcticus* | *A. antarcticus* southeastern AUS | Australian Museum | AM 45505 |
| *A. antarcticus* | *A. antarcticus* southeastern AUS | Australian Museum | AM 47543 |
| *A. antarcticus* | *A. antarcticus* southeastern AUS | Australian Museum | AM 48113 |
| *A. antarcticus* | *A. antarcticus* southeastern AUS | Australian Museum | AM 74297 |
| *A. antarcticus* | *A. antarcticus* southeastern AUS | Australian Museum | AM 74299 |
| *A. antarcticus* | *A. antarcticus* southeastern AUS | Australian Museum | AM 74300 |
| *A. antarcticus* | *A. antarcticus* southeastern AUS | Australian Museum | AM 74301 |
| *A. antarcticus* | *A. antarcticus* southeastern AUS | Australian Museum | AM 74311 |
| *A. antarcticus* | *A. antarcticus* southeastern AUS | Australian Museum | AM 80906 |
| *A. antarcticus* | *A. antarcticus* southeastern AUS | Australian Museum | AM (no tag#) |
| *A. antarcticus* | *A. antarcticus* southeastern AUS | Australian Museum | AM (no tag#) |
| *A. antarcticus* | *A. antarcticus* southeastern AUS | Australian Museum | AM (no tag#) |
| *A. antarcticus* | *A. antarcticus* southeastern AUS | Australian Museum | AM R-13-216 |
| *A. antarcticus* | *A. antarcticus* southeastern AUS | Australian Museum | AM R-13-217 |
| *A. antarcticus* | *A. antarcticus* southeastern AUS | Australian Museum | AM R-13-218 |
| *A. antarcticus* | *A. antarcticus* southeastern AUS | Australian Museum | AM R-13-428 |
| *A. antarcticus* | *A. antarcticus* southeastern AUS | Australian Museum | AM R-13-548 |
| *A. antarcticus* | *A. antarcticus* southeastern AUS | Australian Museum | AM R-13-643 |
| *A. antarcticus* | *A. antarcticus* southeastern AUS | Australian Museum | AM R-13-644 |
| *A. antarcticus* | *A. antarcticus* southeastern AUS | Australian Museum | AM R-13-645 |
| *A. antarcticus* | *A. antarcticus* southeastern AUS | Australian Museum | AM R-13-684 |
| *A. antarcticus* | *A. antarcticus* southeastern AUS | Australian Museum | AM (no tag#) |
| *A. antarcticus* | *A. antarcticus* southeastern AUS | Australian Museum | AM (no tag#) |
| *A. antarcticus* | *A. antarcticus* southeastern AUS | Australian Museum | AM (no tag#) |
| *A. antarcticus* | *A. antarcticus* southeastern AUS | Australian Museum | AM (no tag#) |
| *A. antarcticus* | *A. antarcticus* southeastern AUS | Australian Museum | AM (no tag#) |
| *A. antarcticus* | *A. antarcticus* southeastern AUS | Australian Museum | AM (no tag#) |
| *A. antarcticus* | *A. antarcticus* southeastern AUS | Australian Museum | AM (no tag#) |
| *A. antarcticus* | *A. antarcticus* southeastern AUS | Bishop Museum | BPBM 717 |
| *A. antarcticus* | *A. antarcticus* southeastern AUS | Bishop Museum | BPBM 1271 |
| *A. antarcticus* | *A. antarcticus* southeastern AUS | California Academy of Sciences | CAS 77761 |
| *A. antarcticus* | *A. antarcticus* southeastern AUS | California Academy of Sciences | CAS 77762 |
| *A. antarcticus* | *A. antarcticus* southeastern AUS | California Academy of Sciences | CAS 77793 |
| *A. antarcticus* | *A. antarcticus* southeastern AUS | California Academy of Sciences | CAS 77794 |
| *A. antarcticus* | *A. antarcticus* southeastern AUS | California Academy of Sciences | CAS 136599 |
| *A. antarcticus* | *A. antarcticus* southeastern AUS | California Academy of Sciences | CAS-SU 7297 |
| *A. antarcticus* | *A. antarcticus* southeastern AUS | Field Museum of Natural History | FMNH 75139 |
| *A. antarcticus* | *A. antarcticus* southeastern AUS | Queensland Museum | QM 215 |
| *A. antarcticus* | *A. antarcticus* southeastern AUS | Queensland Museum | QM 516 |
| *A. antarcticus* | *A. antarcticus* southeastern AUS | Queensland Museum | QM 745 |
| *A. antarcticus* | *A. antarcticus* southeastern AUS | Queensland Museum | QM 1175 |
| *A. antarcticus* | *A. antarcticus* southeastern AUS | Queensland Museum | QM 1176 |
| *A. antarcticus* | *A. antarcticus* southeastern AUS | Queensland Museum | QM 1177 |
| *A. antarcticus* | *A. antarcticus* southeastern AUS | Queensland Museum | QM 1265 |
| *A. antarcticus* | *A. antarcticus* southeastern AUS | Queensland Museum | QM 1500 |
| *A. antarcticus* | *A. antarcticus* southeastern AUS | Queensland Museum | QM 1905 |
| *A. antarcticus* | *A. antarcticus* southeastern AUS | Queensland Museum | QM 1937 |
| *A. antarcticus* | *A. antarcticus* southeastern AUS | Queensland Museum | QM 2065 |
| *A. antarcticus* | *A. antarcticus* southeastern AUS | Queensland Museum | QM 3116 |
| *A. antarcticus* | *A. antarcticus* southeastern AUS | Queensland Museum | QM 3200 |
| *A. antarcticus* | *A. antarcticus* southeastern AUS | Queensland Museum | QM 3361 |
| *A. antarcticus* | *A. antarcticus* southeastern AUS | Queensland Museum | QM 3568 |
| *A. antarcticus* | *A. antarcticus* southeastern AUS | Queensland Museum | QM 3802 |
| *A. antarcticus* | *A. antarcticus* southeastern AUS | Queensland Museum | QM 3827 |
| *A. antarcticus* | *A. antarcticus* southeastern AUS | Queensland Museum | QM 4015 |
| *A. antarcticus* | *A. antarcticus* southeastern AUS | Queensland Museum | QM 4023 |
| *A. antarcticus* | *A. antarcticus* southeastern AUS | Queensland Museum | QM 4145 |
| *A. antarcticus* | *A. antarcticus* southeastern AUS | Queensland Museum | QM 4162 |
| *A. antarcticus* | *A. antarcticus* southeastern AUS | Queensland Museum | QM 4463 |
| *A. antarcticus* | *A. antarcticus* southeastern AUS | Queensland Museum | QM 4618 |
| *A. antarcticus* | *A. antarcticus* southeastern AUS | Queensland Museum | QM 4717 |
| *A. antarcticus* | *A. antarcticus* southeastern AUS | Queensland Museum | QM 4795 |
| *A. antarcticus* | *A. antarcticus* southeastern AUS | Queensland Museum | QM 4970 |
| *A. antarcticus* | *A. antarcticus* southeastern AUS | Queensland Museum | QM 5265 |
| *A. antarcticus* | *A. antarcticus* southeastern AUS | Queensland Museum | QM 5424 |
| *A. antarcticus* | *A. antarcticus* southeastern AUS | Queensland Museum | QM 5697 |
| *A. antarcticus* | *A. antarcticus* southeastern AUS | Queensland Museum | QM 5698 |
| *A. antarcticus* | *A. antarcticus* southeastern AUS | Queensland Museum | QM 5744 |
| *A. antarcticus* | *A. antarcticus* southeastern AUS | Queensland Museum | QM 6158 |
| *A. antarcticus* | *A. antarcticus* southeastern AUS | Queensland Museum | QM 6484 |
| *A. antarcticus* | *A. antarcticus* southeastern AUS | Queensland Museum | QM 6492 |
| *A. antarcticus* | *A. antarcticus* southeastern AUS | Queensland Museum | QM 6519 |
| *A. antarcticus* | *A. antarcticus* southeastern AUS | Queensland Museum | QM 6527 |
| *A. antarcticus* | *A. antarcticus* southeastern AUS | Queensland Museum | QM 6610 |
| *A. antarcticus* | *A. antarcticus* southeastern AUS | Queensland Museum | QM 6779 |
| *A. antarcticus* | *A. antarcticus* southeastern AUS | Queensland Museum | QM 6941 |
| *A. antarcticus* | *A. antarcticus* southeastern AUS | Queensland Museum | QM 7021 |
| *A. antarcticus* | *A. antarcticus* southeastern AUS | Queensland Museum | QM 7047 |
| *A. antarcticus* | *A. antarcticus* southeastern AUS | Queensland Museum | QM 7048 |
| *A. antarcticus* | *A. antarcticus* southeastern AUS | Queensland Museum | QM 7208 |
| *A. antarcticus* | *A. antarcticus* southeastern AUS | Queensland Museum | QM 7210 |
| *A. antarcticus* | *A. antarcticus* southeastern AUS | Queensland Museum | QM 7571 |
| *A. antarcticus* | *A. antarcticus* southeastern AUS | Queensland Museum | QM 7638 |
| *A. antarcticus* | *A. antarcticus* southeastern AUS | Queensland Museum | QM 7740 |
| *A. antarcticus* | *A. antarcticus* southeastern AUS | Queensland Museum | QM 7805 |
| *A. antarcticus* | *A. antarcticus* southeastern AUS | Queensland Museum | QM 7873 |
| *A. antarcticus* | *A. antarcticus* southeastern AUS | Queensland Museum | QM 8006 |
| *A. antarcticus* | *A. antarcticus* southeastern AUS | Queensland Museum | QM 8104 |
| *A. antarcticus* | *A. antarcticus* southeastern AUS | Queensland Museum | QM 8105 |
| *A. antarcticus* | *A. antarcticus* southeastern AUS | Queensland Museum | QM 8191 |
| *A. antarcticus* | *A. antarcticus* southeastern AUS | Queensland Museum | QM 8200 |
| *A. antarcticus* | *A. antarcticus* southeastern AUS | Queensland Museum | QM 8512 |
| *A. antarcticus* | *A. antarcticus* southeastern AUS | Queensland Museum | QM 8637 |
| *A. antarcticus* | *A. antarcticus* southeastern AUS | Queensland Museum | QM 8737 |
| *A. antarcticus* | *A. antarcticus* southeastern AUS | Queensland Museum | QM 9755 |
| *A. antarcticus* | *A. antarcticus* southeastern AUS | Queensland Museum | QM 10708 |
| *A. antarcticus* | *A. antarcticus* southeastern AUS | Queensland Museum | QM 10888 |
| *A. antarcticus* | *A. antarcticus* southeastern AUS | Queensland Museum | QM 11279 |
| *A. antarcticus* | *A. antarcticus* southeastern AUS | Queensland Museum | QM 11568 |
| *A. antarcticus* | *A. antarcticus* southeastern AUS | Queensland Museum | QM 11622 |
| *A. antarcticus* | *A. antarcticus* southeastern AUS | Queensland Museum | QM 11637 |
| *A. antarcticus* | *A. antarcticus* southeastern AUS | Queensland Museum | QM 12398 |
| *A. antarcticus* | *A. antarcticus* southeastern AUS | Queensland Museum | QM 13260 |
| *A. antarcticus* | *A. antarcticus* southeastern AUS | Queensland Museum | QM 13277 |
| *A. antarcticus* | *A. antarcticus* southeastern AUS | Queensland Museum | QM 13452 |
| *A. antarcticus* | *A. antarcticus* southeastern AUS | Queensland Museum | QM 13453 |
| *A. antarcticus* | *A. antarcticus* southeastern AUS | Queensland Museum | QM 13454 |
| *A. antarcticus* | *A. antarcticus* southeastern AUS | Queensland Museum | QM 13455 |
| *A. antarcticus* | *A. antarcticus* southeastern AUS | Queensland Museum | QM 13456 |
| *A. antarcticus* | *A. antarcticus* southeastern AUS | Queensland Museum | QM 13457 |
| *A. antarcticus* | *A. antarcticus* southeastern AUS | Queensland Museum | QM 13458 |
| *A. antarcticus* | *A. antarcticus* southeastern AUS | Queensland Museum | QM 13459 |
| *A. antarcticus* | *A. antarcticus* southeastern AUS | Queensland Museum | QM 13460 |
| *A. antarcticus* | *A. antarcticus* southeastern AUS | Queensland Museum | QM 13679 |
| *A. antarcticus* | *A. antarcticus* southeastern AUS | Queensland Museum | QM 13680 |
| *A. antarcticus* | *A. antarcticus* southeastern AUS | Queensland Museum | QM 14303 |
| *A. antarcticus* | *A. antarcticus* southeastern AUS | Queensland Museum | QM 14750 |
| *A. antarcticus* | *A. antarcticus* southeastern AUS | Queensland Museum | QM 17570 |
| *A. antarcticus* | *A. antarcticus* southeastern AUS | Queensland Museum | QM 17993 |
| *A. antarcticus* | *A. antarcticus* southeastern AUS | Queensland Museum | QM 17994 |
| *A. antarcticus* | *A. antarcticus* southeastern AUS | Queensland Museum | QM 19050 |
| *A. antarcticus* | *A. antarcticus* southeastern AUS | Queensland Museum | QM 19052 |
| *A. antarcticus* | *A. antarcticus* southeastern AUS | Queensland Museum | QM 20236 |
| *A. antarcticus* | *A. antarcticus* southeastern AUS | Queensland Museum | QM 20237 |
| *A. antarcticus* | *A. antarcticus* southeastern AUS | Queensland Museum | QM 20238 |
| *A. antarcticus* | *A. antarcticus* southeastern AUS | Queensland Museum | QM 20296 |
| *A. antarcticus* | *A. antarcticus* southeastern AUS | Queensland Museum | QM 20616 |
| *A. antarcticus* | *A. antarcticus* southeastern AUS | Queensland Museum | QM 20657 |
| *A. antarcticus* | *A. antarcticus* southeastern AUS | Queensland Museum | QM 20659 |
| *A. antarcticus* | *A. antarcticus* southeastern AUS | Queensland Museum | QM 20661 |
| *A. antarcticus* | *A. antarcticus* southeastern AUS | Queensland Museum | QM 20662 |
| *A. antarcticus* | *A. antarcticus* southeastern AUS | Queensland Museum | QM 20663 |
| *A. antarcticus* | *A. antarcticus* southeastern AUS | Queensland Museum | QM 21935 |
| *A. antarcticus* | *A. antarcticus* southeastern AUS | Queensland Museum | QM 22661 |
| *A. antarcticus* | *A. antarcticus* southeastern AUS | Queensland Museum | QM 23209 |
| *A. antarcticus* | *A. antarcticus* southeastern AUS | Queensland Museum | QM 23647 |
| *A. antarcticus* | *A. antarcticus* southeastern AUS | Queensland Museum | QM 23690 |
| *A. antarcticus* | *A. antarcticus* southeastern AUS | Queensland Museum | QM 23709 |
| *A. antarcticus* | *A. antarcticus* southeastern AUS | Queensland Museum | QM 23716 |
| *A. antarcticus* | *A. antarcticus* southeastern AUS | Queensland Museum | QM 23754 |
| *A. antarcticus* | *A. antarcticus* southeastern AUS | Queensland Museum | QM 23755 |
| *A. antarcticus* | *A. antarcticus* southeastern AUS | Queensland Museum | QM 23758 |
| *A. antarcticus* | *A. antarcticus* southeastern AUS | Queensland Museum | QM 24141 |
| *A. antarcticus* | *A. antarcticus* southeastern AUS | Queensland Museum | QM 24328 |
| *A. antarcticus* | *A. antarcticus* southeastern AUS | Queensland Museum | QM 24468 |
| *A. antarcticus* | *A. antarcticus* southeastern AUS | Queensland Museum | QM 24960 |
| *A. antarcticus* | *A. antarcticus* southeastern AUS | Queensland Museum | QM 25436 |
| *A. antarcticus* | *A. antarcticus* southeastern AUS | Queensland Museum | QM 28398 |
| *A. antarcticus* | *A. antarcticus* southeastern AUS | Queensland Museum | QM 28764 |
| *A. antarcticus* | *A. antarcticus* southeastern AUS | Queensland Museum | QM 28779 |
| *A. antarcticus* | *A. antarcticus* southeastern AUS | Queensland Museum | QM 28866 |
| *A. antarcticus* | *A. antarcticus* southeastern AUS | Queensland Museum | QM 28867 |
| *A. antarcticus* | *A. antarcticus* southeastern AUS | Queensland Museum | QM 28868 |
| *A. antarcticus* | *A. antarcticus* southeastern AUS | Queensland Museum | QM 30433 |
| *A. pyrrhus* | *A. antarcticus* southeastern AUS | Queensland Museum | QM 31646 |
| *A. antarcticus* | *A. antarcticus* southeastern AUS | Queensland Museum | QM 31980 |
| *A. antarcticus* | *A. antarcticus* southeastern AUS | Queensland Museum | QM 31981 |
| *A. antarcticus* | *A. antarcticus* southeastern AUS | Queensland Museum | QM 31998 |
| *A. antarcticus* | *A. antarcticus* southeastern AUS | Queensland Museum | QM 31999 |
| *A. antarcticus* | *A. antarcticus* southeastern AUS | Queensland Museum | QM 32001 |
| *A. antarcticus* | *A. antarcticus* southeastern AUS | Queensland Museum | QM 32267 |
| *A. antarcticus* | *A. antarcticus* southeastern AUS | Queensland Museum | QM 32298 |
| *A. antarcticus* | *A. antarcticus* southeastern AUS | Queensland Museum | QM 33884 |
| *A. antarcticus* | *A. antarcticus* southeastern AUS | Queensland Museum | QM 34314 |
| *A. antarcticus* | *A. antarcticus* southeastern AUS | Queensland Museum | QM 34320 |
| *A. antarcticus* | *A. antarcticus* southeastern AUS | Queensland Museum | QM 34595 |
| *A. antarcticus* | *A. antarcticus* southeastern AUS | Queensland Museum | QM 35133 |
| *A. antarcticus* | *A. antarcticus* southeastern AUS | Queensland Museum | QM 35403 |
| *A. antarcticus* | *A. antarcticus* southeastern AUS | Queensland Museum | QM (no tag#) |
| *A. antarcticus* | *A. antarcticus* southeastern AUS | University of Michigan Museum of Zoology | UMMZ 65649 |
| *A. antarcticus* | *A. antarcticus* southeastern AUS | University of Michigan Museum of Zoology | UMMZ 83504 |
| *A. antarcticus* | *A. antarcticus* southeastern AUS | University of Michigan Museum of Zoology | UMMZ 83505 |
| *A. antarcticus* | *A. antarcticus* southeastern AUS | United States National Museum | USNM 11032 |
| *A. antarcticus* | *A. antarcticus* southeastern AUS | United States National Museum | USNM 17880 |
| *A. antarcticus* | *A. antarcticus* southeastern AUS | United States National Museum | USNM 56002 |
| *A. antarcticus* | *A. antarcticus* southeastern AUS | United States National Museum | USNM 63123 |
| *A. antarcticus* | *A. antarcticus* southwestern AUS | Australia Museum | AM 4962 |
| *A. antarcticus* | *A. antarcticus* southwestern AUS | Australia Museum | AM 8370 |
| *A. antarcticus* | *A. antarcticus* southwestern AUS | Australia Museum | AM 51661 |
| *A. antarcticus* | *A. antarcticus* southwestern AUS | Western Australia Museum | WAM 10393 |
| *A. antarcticus* | *A. antarcticus* southwestern AUS | Western Australia Museum | WAM 113756 |
| *A. antarcticus* | *A. antarcticus* southwestern AUS | Western Australia Museum | WAM 12267 |
| *A. antarcticus* | *A. antarcticus* southwestern AUS | Western Australia Museum | WAM 13693 |
| *A. antarcticus* | *A. antarcticus* southwestern AUS | Western Australia Museum | WAM 1523 |
| *A. antarcticus* | *A. antarcticus* southwestern AUS | Western Australia Museum | WAM 2160 |
| *A. antarcticus* | *A. antarcticus* southwestern AUS | Western Australia Museum | WAM 22332 |
| *A. antarcticus* | *A. antarcticus* southwestern AUS | Western Australia Museum | WAM 248 |
| *A. antarcticus* | *A. antarcticus* southwestern AUS | Western Australia Museum | WAM 25080 |
| *A. antarcticus* | *A. antarcticus* southwestern AUS | Western Australia Museum | WAM 26351 |
| *A. antarcticus* | *A. antarcticus* southwestern AUS | Western Australia Museum | WAM 26688 |
| *A. antarcticus* | *A. antarcticus* southwestern AUS | Western Australia Museum | WAM 26689 |
| *A. antarcticus* | *A. antarcticus* southwestern AUS | Western Australia Museum | WAM 26803 |
| *A. antarcticus* | *A. antarcticus* southwestern AUS | Western Australia Museum | WAM 28096 |
| *A. antarcticus* | *A. antarcticus* southwestern AUS | Western Australia Museum | WAM 28160 |
| *A. antarcticus* | *A. antarcticus* southwestern AUS | Western Australia Museum | WAM 2820 |
| *A. antarcticus* | *A. antarcticus* southwestern AUS | Western Australia Museum | WAM 34068 |
| *A. antarcticus* | *A. antarcticus* southwestern AUS | Western Australia Museum | WAM 37720 |
| *A. antarcticus* | *A. antarcticus* southwestern AUS | Western Australia Museum | WAM 40197 |
| *A. antarcticus* | *A. antarcticus* southwestern AUS | Western Australia Museum | WAM 44975 |
| *A. antarcticus* | *A. antarcticus* southwestern AUS | Western Australia Museum | WAM 51814 |
| *A. antarcticus* | *A. antarcticus* southwestern AUS | Western Australia Museum | WAM 53096 |
| *A. antarcticus* | *A. antarcticus* southwestern AUS | Western Australia Museum | WAM 5619 |
| *A. antarcticus* | *A. antarcticus* southwestern AUS | Western Australia Museum | WAM 58779 |
| *A. antarcticus* | *A. antarcticus* southwestern AUS | Western Australia Museum | WAM 5948 |
| *A. antarcticus* | *A. antarcticus* southwestern AUS | Western Australia Museum | WAM 64698 |
| *A. antarcticus* | *A. antarcticus* southwestern AUS | Western Australia Museum | WAM 7402 |
| *A. antarcticus* | *A. antarcticus* southwestern AUS | Western Australia Museum | WAM 77849 |
| *A. antarcticus* | *A. antarcticus* southwestern AUS | Western Australia Museum | WAM 78980 |
| *A. antarcticus* | *A. antarcticus* southwestern AUS | Western Australia Museum | WAM 82730 |
| *A. antarcticus* | *A. antarcticus* southwestern AUS | Western Australia Museum | WAM 9576 |
| *A. antarcticus* | *A. antarcticus* southwestern AUS | Western Australia Museum | WAM 9643 |
| *A. antarcticus* | *A. praelongus* northern AUS | Australian Museum | AM 1547 |
| *A. antarcticus* | *A. praelongus* northern AUS | Australian Museum | AM 3785 |
| *A. antarcticus* | *A. praelongus* northern AUS | Australian Museum | AM 3785 |
| *A. antarcticus* | *A. praelongus* northern AUS | Australian Museum | AM 9695 |
| *A. antarcticus* | *A. praelongus* northern AUS | Australian Museum | AM 10218 |
| *A. antarcticus* | *A. praelongus* northern AUS | Australian Museum | AM 10219 |
| *A. pyrrhus* | *A. praelongus* northern AUS | Australian Museum | AM 10859 |
| *A. antarcticus* | *A. praelongus* northern AUS | Australian Museum | AM 11023 |
| *A. antarcticus* | *A. praelongus* northern AUS | Australian Museum | AM 11024 |
| *A. antarcticus* | *A. praelongus* northern AUS | Australian Museum | AM 12438 |
| *A. antarcticus* | *A. praelongus* northern AUS | Australian Museum | AM 12439 |
| *A. antarcticus* | *A. praelongus* northern AUS | Australian Museum | AM 12539 |
| *A. antarcticus* | *A. praelongus* northern AUS | Australian Museum | AM 12552 |
| *A. antarcticus* | *A. praelongus* northern AUS | Australian Museum | AM 13787 |
| *A. antarcticus* | *A. praelongus* northern AUS | Australian Museum | AM 14924 |
| *A. antarcticus* | *A. praelongus* northern AUS | Australian Museum | AM 16733 |
| *A. antarcticus* | *A. praelongus* northern AUS | Australian Museum | AM 16863 |
| *A. antarcticus* | *A. praelongus* northern AUS | Australian Museum | AM 18948 |
| *A. antarcticus* | *A. praelongus* northern AUS | Australian Museum | AM 26274 |
| *A. antarcticus* | *A. praelongus* northern AUS | Australian Museum | AM 33216 |
| *A. pyrrhus* | *A. praelongus* northern AUS | Australian Museum | AM 47476 |
| *A. antarcticus* | *A. praelongus* northern AUS | Australian Museum | AM 57292 |
| *A. antarcticus* | *A. praelongus* northern AUS | Australian Museum | AM 58551 |
| *A. antarcticus* | *A. praelongus* northern AUS | Australian Museum | AM 58552 |
| *A. pyrrhus* | *A. praelongus* northern AUS | Australian Museum | AM 72612 |
| *A. antarcticus* | *A. praelongus* northern AUS | Australian Museum | AM 74302 |
| *A. antarcticus* | *A. praelongus* northern AUS | Australian Museum | AM 74312 |
| *A. antarcticus* | *A. praelongus* northern AUS | Australian Museum | AM 75034 |
| *A. antarcticus* | *A. praelongus* northern AUS | Australian Museum | AM 76968 |
| *A. antarcticus* | *A. praelongus* northern AUS | Australian Museum | AM (no tag#) |
| *A. antarcticus* | *A. praelongus* northern AUS | Field Museum of Natural History | FMNH 29019 |
| *A. antarcticus* | *A. praelongus* northern AUS | Field Museum of Natural History | FMNH 97035 |
| *A. antarcticus* | *A. praelongus* northern AUS | Northern Territory Museum | NTM 1160 |
| *A. antarcticus* | *A. praelongus* northern AUS | Northern Territory Museum | NTM 1200 |
| *A. antarcticus* | *A. praelongus* northern AUS | Northern Territory Museum | NTM 1201 |
| *A. antarcticus* | *A. praelongus* northern AUS | Northern Territory Museum | NTM 12898 |
| *A. praelongus* | *A. praelongus* northern AUS | Northern Territory Museum | NTM 16639 |
| *A. antarcticus* | *A. praelongus* northern AUS | Northern Territory Museum | NTM 16640 |
| *A. praelongus* | *A. praelongus* northern AUS | Northern Territory Museum | NTM 16707 |
| *A. praelongus* | *A. praelongus* northern AUS | Northern Territory Museum | NTM 17878 |
| *A. antarcticus* | *A. praelongus* northern AUS | Northern Territory Museum | NTM 17879 |
| *A. antarcticus* | *A. praelongus* northern AUS | Northern Territory Museum | NTM 17880 |
| *A. antarcticus* | *A. praelongus* northern AUS | Northern Territory Museum | NTM 17881 |
| *A. antarcticus* | *A. praelongus* northern AUS | Northern Territory Museum | NTM 17882 |
| *A. praelongus* | *A. praelongus* northern AUS | Northern Territory Museum | NTM 17916 |
| *A. praelongus* | *A. praelongus* northern AUS | Northern Territory Museum | NTM 17918 |
| *A. antarcticus* | *A. praelongus* northern AUS | Northern Territory Museum | NTM 2968 |
| *A. antarcticus* | *A. praelongus* northern AUS | Northern Territory Museum | NTM 31209 |
| *A. antarcticus* | *A. praelongus* northern AUS | Northern Territory Museum | NTM 31210 |
| *A. antarcticus* | *A. praelongus* northern AUS | Northern Territory Museum | NTM 31211 |
| *A. antarcticus* | *A. praelongus* northern AUS | Northern Territory Museum | NTM 31212 |
| *A. antarcticus* | *A. praelongus* northern AUS | Northern Territory Museum | NTM 31213 |
| *A. antarcticus* | *A. praelongus* northern AUS | Northern Territory Museum | NTM 31214 |
| *A. antarcticus* | *A. praelongus* northern AUS | Northern Territory Museum | NTM 3353 |
| *A. antarcticus* | *A. praelongus* northern AUS | Northern Territory Museum | NTM 3354 |
| *A. antarcticus* | *A. praelongus* northern AUS | Northern Territory Museum | NTM 3677 |
| *A. antarcticus* | *A. praelongus* northern AUS | Northern Territory Museum | NTM 4265 |
| *A. antarcticus* | *A. praelongus* northern AUS | Northern Territory Museum | NTM 4266 |
| *A. antarcticus* | *A. praelongus* northern AUS | Northern Territory Museum | NTM 5201 |
| *A. antarcticus* | *A. praelongus* northern AUS | Northern Territory Museum | NTM 5248 |
| *A. antarcticus* | *A. praelongus* northern AUS | Northern Territory Museum | NTM 6510 |
| *A. antarcticus* | *A. praelongus* northern AUS | Northern Territory Museum | NTM 6893 |
| *A. antarcticus* | *A. praelongus* northern AUS | Northern Territory Museum | NTM 7068 |
| *A. antarcticus* | *A. praelongus* northern AUS | Northern Territory Museum | NTM 7069 |
| *A. antarcticus* | *A. praelongus* northern AUS | Northern Territory Museum | NTM 8191 |
| *A. antarcticus* | *A. praelongus* northern AUS | Northern Territory Museum | NTM 8193 |
| *A. antarcticus* | *A. praelongus* northern AUS | Northern Territory Museum | NTM 8194 |
| *A. antarcticus* | *A. praelongus* northern AUS | Northern Territory Museum | NTM 8195 |
| *A. antarcticus* | *A. praelongus* northern AUS | Northern Territory Museum | NTM 8196 |
| *A. antarcticus* | *A. praelongus* northern AUS | Northern Territory Museum | NTM 8197 |
| *A. antarcticus* | *A. praelongus* northern AUS | Northern Territory Museum | NTM 8200 |
| *A. antarcticus* | *A. praelongus* northern AUS | Northern Territory Museum | NTM 8201 |
| *A. antarcticus* | *A. praelongus* northern AUS | Northern Territory Museum | NTM 8378 |
| *A. antarcticus* | *A. praelongus* northern AUS | Northern Territory Museum | NTM 8610 |
| *A. antarcticus* | *A. praelongus* northern AUS | Northern Territory Museum | NTM 8611 |
| *A. antarcticus* | *A. praelongus* northern AUS | Northern Territory Museum | NTM 9327 |
| *A. antarcticus* | *A. praelongus* northern AUS | Northern Territory Museum | NTM 9328 |
| *A. antarcticus* | *A. praelongus* northern AUS | Northern Territory Museum | NTM 9684 |
| *A. antarcticus* | *A. praelongus* northern AUS | Northern Territory Museum | NTM 9685 |
| *A. antarcticus* | *A. praelongus* northern AUS | Northern Territory Museum | NTM 9686 |
| *A. antarcticus* | *A. praelongus* northern AUS | Northern Territory Museum | NTM 9697 |
| *A. antarcticus* | *A. praelongus* northern AUS | Northern Territory Museum | NTM 9698 |
| *A. antarcticus* | *A. praelongus* northern AUS | Northern Territory Museum | NTM 9699 |
| *A. antarcticus* | *A. praelongus* northern AUS | Northern Territory Museum | NTM 9722 |
| *A. antarcticus* | *A. praelongus* northern AUS | Northern Territory Museum | NTM 9723 |
| *A. antarcticus* | *A. praelongus* northern AUS | Northern Territory Museum | NTM 9724 |
| *A. antarcticus* | *A. praelongus* northern AUS | Northern Territory Museum | NTM 9725 |
| *A. antarcticus* | *A. praelongus* northern AUS | Northern Territory Museum | NTM 9726 |
| *A. antarcticus* | *A. praelongus* northern AUS | Northern Territory Museum | NTM 9727 |
| *A. antarcticus* | *A. praelongus* northern AUS | Northern Territory Museum | NTM 9758 |
| *A. antarcticus* | *A. praelongus* northern AUS | Northern Territory Museum | NTM 9765 |
| *A. antarcticus* | *A. praelongus* northern AUS | Northern Territory Museum | NTM 9829 |
| *A. antarcticus* | *A. praelongus* northern AUS | Northern Territory Museum | NTM 9830 |
| *A. antarcticus* | *A. praelongus* northern AUS | Northern Territory Museum | NTM 9991 |
| *A. antarcticus* | *A. praelongus* northern AUS | Northern Territory Museum | NTM 9992 |
| *A. antarcticus* | *A. praelongus* northern AUS | University of Michigan Museum of Zoology | UMMZ 65453 |
| *A. antarcticus* | *A. praelongus* northern AUS | University of Michigan Museum of Zoology | UMMZ 203473 |
| *A. praelongus* | *A. praelongus* northern AUS | United States National Museum | UMMZ 128449 |
| *A. praelongus* | *A. praelongus* northern AUS | United States National Museum | UMMZ 128476 |
| *A. praelongus* | *A. praelongus* northern AUS | United States National Museum | UMMZ 128477 |
| *A. antarcticus* | *A. praelongus* northern AUS | Western Australia Museum | WAM (no tag#) |
| *A. antarcticus* | *A. praelongus* northern AUS | Western Australia Museum | WAM 102060 |
| *A. antarcticus* | *A. praelongus* northern AUS | Western Australia Museum | WAM 11241 |
| *A. antarcticus* | *A. praelongus* northern AUS | Western Australia Museum | WAM 113130 |
| *A. antarcticus* | *A. praelongus* northern AUS | Western Australia Museum | WAM 21519 |
| *A. antarcticus* | *A. praelongus* northern AUS | Western Australia Museum | WAM 28099 |
| *A. antarcticus* | *A. praelongus* northern AUS | Western Australia Museum | WAM 34079 |
| *A. antarcticus* | *A. praelongus* northern AUS | Western Australia Museum | WAM 37761 |
| *A. antarcticus* | *A. praelongus* northern AUS | Western Australia Museum | WAM 37762 |
| *A. antarcticus* | *A. praelongus* northern AUS | Western Australia Museum | WAM 37763 |
| *A. antarcticus* | *A. praelongus* northern AUS | Western Australia Museum | WAM 37764 |
| *A. antarcticus* | *A. praelongus* northern AUS | Western Australia Museum | WAM 41457 |
| *A. antarcticus* | *A. praelongus* northern AUS | Western Australia Museum | WAM 609 |
| *A. antarcticus* | *A. praelongus* northern AUS | Western Australia Museum | WAM 81245 |
| *A. antarcticus* | *A. praelongus* northern AUS | Western Australia Museum | WAM 87934 |
| *A. pyrrhus* | *A. pyrrhus* | Australian Museum | AM 47477 |
| *A. pyrrhus* | *A. pyrrhus* | Australian Museum | AM 47478 |
| *A. pyrrhus* | *A. pyrrhus* | Northern Territory Museum | NTM 15945 |
| *A. pyrrhus* | *A. pyrrhus* | Northern Territory Museum | NTM 1957 |
| *A. pyrrhus* | *A. pyrrhus* | Northern Territory Museum | NTM 310 |
| *A. pyrrhus* | *A. pyrrhus* | Northern Territory Museum | NTM 31216 |
| *A. pyrrhus* | *A. pyrrhus* | Northern Territory Museum | NTM 31221 |
| *A. pyrrhus* | *A. pyrrhus* | Northern Territory Museum | NTM 31225 |
| *A. pyrrhus* | *A. pyrrhus* | Northern Territory Museum | NTM 31226 |
| *A. pyrrhus* | *A. pyrrhus* | Northern Territory Museum | NTM 7189 |
| *A. pyrrhus* | *A. pyrrhus* | Northern Territory Museum | NTM 8025 |
| *A. pyrrhus* | *A. pyrrhus* | Northern Territory Museum | NTM 9974 |
| *A. pyrrhus* | *A. pyrrhus* | Western Australia Museum | WAM 10033 |
| *A. pyrrhus* | *A. pyrrhus* | Western Australia Museum | WAM 104357 |
| *A. pyrrhus* | *A. pyrrhus* | Western Australia Museum | WAM 15101 |
| *A. pyrrhus* | *A. pyrrhus* | Western Australia Museum | WAM 1767 |
| *A. antarcticus* | *A. pyrrhus* | Western Australia Museum | WAM 19804 |
| *A. pyrrhus* | *A. pyrrhus* | Western Australia Museum | WAM 2130 |
| *A. pyrrhus* | *A. pyrrhus* | Western Australia Museum | WAM 2138 |
| *A. pyrrhus* | *A. pyrrhus* | Western Australia Museum | WAM 28097 |
| *A. pyrrhus* | *A. pyrrhus* | Western Australia Museum | WAM 28098 |
| *A. pyrrhus* | *A. pyrrhus* | Western Australia Museum | WAM 28101 |
| *A. pyrrhus* | *A. pyrrhus* | Western Australia Museum | WAM 28102 |
| *A. pyrrhus* | *A. pyrrhus* | Western Australia Museum | WAM 28105 |
| *A. pyrrhus* | *A. pyrrhus* | Western Australia Museum | WAM 28106 |
| *A. pyrrhus* | *A. pyrrhus* | Western Australia Museum | WAM 28107 |
| *A. pyrrhus* | *A. pyrrhus* | Western Australia Museum | WAM 46079 |
| *A. pyrrhus* | *A. pyrrhus* | Western Australia Museum | WAM 49985 |
| *A. pyrrhus* | *A. pyrrhus* | Western Australia Museum | WAM 70699 |
| *A. pyrrhus* | *A. pyrrhus* | Western Australia Museum | WAM 70700 |
| *A. pyrrhus* | *A. pyrrhus* | Western Australia Museum | WAM 70701 |
| *A. pyrrhus* | *A. pyrrhus* | Western Australia Museum | WAM 71601 |
| *A. pyrrhus* | *A. pyrrhus* | Western Australia Museum | WAM 73134 |
| *A. pyrrhus* | *A. pyrrhus* | Western Australia Museum | WAM 75012 |
| *A. pyrrhus* | *A. pyrrhus* | Western Australia Museum | WAM 7868 |
| *A. pyrrhus* | *A. pyrrhus* | Western Australia Museum | WAM 79006 |
| *A. pyrrhus* | *A. pyrrhus* | Western Australia Museum | WAM 79008 |
| *A. pyrrhus* | *A. pyrrhus* | Western Australia Museum | WAM 79030 |
| *A. pyrrhus* | *A. pyrrhus* | Western Australia Museum | WAM 79138 |
| *A. pyrrhus* | *A. pyrrhus* | Western Australia Museum | WAM 79139 |
| *A. pyrrhus* | *A. pyrrhus* | Western Australia Museum | WAM 81846 |
| *A. pyrrhus* | *A. pyrrhus* | Western Australia Museum | WAM 85116 |
| *A. pyrrhus* | *A. pyrrhus* | Western Australia Museum | WAM 91671 |
| *A. pyrrhus* | *A. pyrrhus* | Western Australia Museum | WAM 91672 |
| *A. pyrrhus* | *A. pyrrhus* | Western Australia Museum | WAM 9756 |
| *A. pyrrhus* | *A. pyrrhus* | Western Australia Museum | WAM 9996 |
